# Engineered human cytokine/antibody fusion proteins expand regulatory T cells and confer autoimmune disease protection

**DOI:** 10.1016/j.celrep.2022.111478

**Published:** 2022-10-18

**Authors:** Derek VanDyke, Marcos Iglesias, Jakub Tomala, Arabella Young, Jennifer Smith, Joseph A. Perry, Edward Gebara, Amy R. Cross, Laurene S. Cheung, Arbor G. Dykema, Brian T. Orcutt-Jahns, Tereza Henclová, Jaroslav Golias, Jared Balolong, Luke M. Tomasovic, David Funda, Aaron S. Meyer, Drew M. Pardoll, Joanna Hester, Fadi Issa, Christopher A. Hunter, Mark S. Anderson, Jeffrey A. Bluestone, Giorgio Raimondi, Jamie B. Spangler

**Affiliations:** 1 Department of Chemical and Biomolecular Engineering, Johns Hopkins University, Baltimore, MD 21218, USA; 2 Translational Tissue Engineering Center, Johns Hopkins University School of Medicine, Baltimore, MD 21231, USA; 3 Vascularized Composite Allotransplantation Laboratory, Department of Plastic and Reconstructive Surgery, Johns Hopkins University School of Medicine, Baltimore, MD 21205, USA; 4 Institute of Biotechnology of the Academy of Sciences of the Czech Republic, Vestec 252 50, Czech Republic; 5 Diabetes Center, University of California San Francisco, San Francisco, CA 94143, USA; 6 Sean N. Parker Autoimmune Research Laboratory, University of California San Francisco, San Francisco, CA 94143, USA; 7 Huntsman Cancer Institute, University of Utah Health Sciences Center, Salt Lake City, UT 84112, USA; 8 Department of Pathology, University of Utah School of Medicine, Salt Lake City, UT 84112, USA; 9 Department of Pathobiology, University of Pennsylvania, Philadelphia, PA 19104, USA; 10 Department of Molecular Microbiology and Immunology, Johns Hopkins Bloomberg School of Public Health, Baltimore, MD 21205, USA; 11 Translational Research Immunology Group, Nuffield Department of Surgical Sciences, University of Oxford, Oxford OX3 9DU, UK; 12 Bloomberg-Kimmel Institute for Cancer Immunotherapy, Johns Hopkins University, Baltimore, MD 21231, USA; 13 Sidney Kimmel Comprehensive Cancer Center, Johns Hopkins University, Baltimore, MD 21231, USA; 14 Department of Bioengineering, Jonsson Comprehensive Cancer Center, Eli and Edythe Broad Center of Regenerative Medicine and Stem Cell Research, University of California, Los Angeles, Los Angeles, CA 90095, USA; 15 Institute of Microbiology of the Academy of Sciences of the Czech Republic, Prague 142 20, Czech Republic; 16 Sonoma Biotherapeutics, South San Francisco, CA 94080, USA; 17 Department of Biomedical Engineering, Johns Hopkins University School of Medicine, Baltimore, MD 21205, USA; 18 Department of Oncology, Johns Hopkins University School of Medicine, Baltimore, MD 21231, USA; 19 Department of Ophthalmology, Johns Hopkins University School of Medicine, Baltimore, MD 21287, USA; 20 Lead contact

## Abstract

Low-dose human interleukin-2 (hIL-2) treatment is used clinically to treat autoimmune disorders due to the cytokine’s preferential expansion of immunosuppressive regulatory T cells (Tregs). However, off-target immune cell activation and short serum half-life limit the clinical potential of IL-2 treatment. Recent work showed that complexes comprising hIL-2 and the anti-hIL-2 antibody F5111 overcome these limitations by preferentially stimulating Tregs over immune effector cells. Although promising, therapeutic translation of this approach is complicated by the need to optimize dosing ratios and by the instability of the cytokine/antibody complex. We leverage structural insights to engineer a single-chain hIL-2/F5111 antibody fusion protein, termed F5111 immunocytokine (IC), which potently and selectively activates and expands Tregs. F5111 IC confers protection in mouse models of colitis and checkpoint inhibitor-induced diabetes mellitus. These results provide a roadmap for IC design and establish a Treg-biased immunotherapy that could be clinically translated for autoimmune disease treatment.

## In brief

VanDyke et al. engineer a single-chain fusion protein composed of the human interleukin-2 cytokine and the anti-cytokine antibody F5111, which induces biased activation and expansion of regulatory T cells *in vitro* and *in vivo*. This protein confers protection in animal models of colitis and immune checkpoint inhibitor-induced diabetes mellitus.

## Graphical Abstract

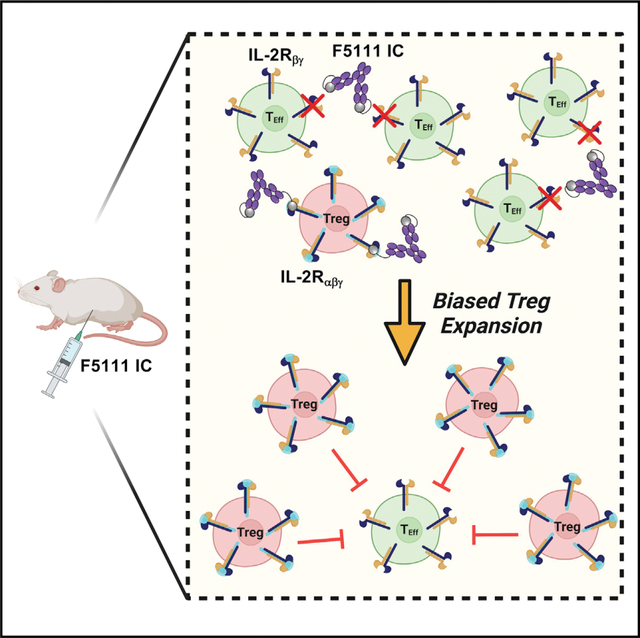

## INTRODUCTION

Interleukin-2 (IL-2) is a pleiotropic cytokine that regulates key homeostatic functions, including proliferation, survival, and activation of both pro-inflammatory immune effector cells (Effs) (e.g., CD4^+^/CD8^+^ effector T cells, natural killer [NK] cells), and anti-inflammatory regulatory T cells (Tregs). IL-2 engages transmembrane receptors to activate signaling through the Janus kinase-signal transducer and activator of transcription (STAT) pathway, which regulates gene expression and functional outcomes (Malek, 2008; Stroud and Wells, 2004; Murray, 2007). IL-2 forms either an intermediate-affinity heterodimeric receptor complex, comprising the IL-2 receptor-β (IL-2Rβ, CD122) and common gamma (γ_c_) (CD132) chains, or a high-affinity heterotrimeric receptor complex, comprising the non-signaling IL-2Rα subunit (also CD25) as well as the IL-2Rβ and γ_c_ chains (Leonard et al., 1984; Liao et al., 2013; Wang et al., 2005). IL-2Rα is highly expressed on Tregs but virtually absent from naive Effs; thus, Tregs are 100-fold more sensitive to IL-2 (Boyman and Sprent, 2012; Sakaguchi et al., 1995; Taniguchi and Minami, 1993). IL-2Rα expression is also induced in activated Effs, albeit at lower levels, and activated Effs may compete with Tregs for extracellular IL-2 (Baecher-Allan et al., 2001; Höfer et al., 2012; Schmidt et al., 2012). Hence, IL-2 promotes both pro- and anti-inflammatory responses, which has made it an attractive, albeit complex, candidate for immunotherapy.

IL-2 first received FDA approval as a pro-inflammatory agent, wherein high doses are administered to treat metastatic cancers (Alva et al., 2016; Rosenberg, 2014; Sim and Radvanyi, 2014). In contrast, low doses of IL-2 have been used to treat autoimmune conditions, such as diabetes, ulcerative colitis, graft-versus-host disease (GVHD), and allograft rejection. However, low-dose IL-2 strategies are limited by the dangerous off-target effects that result from activation of Effs and by the short serum half-life of IL-2.

Several approaches have been explored to overcome the limitations of IL-2 therapy by biasing cytokine activity (Hernandez et al., 2022), including the design of IL-2 muteins (Peterson et al., 2018; Carmenate et al., 2018; Khoryati et al., 2020; Glassman et al., 2021), PEGylated IL-2 variants (Charych et al., 2016; Dixit et al., 2021; Zhang et al., 2021), and IL-2/IL-2Rα fusion proteins (Hernandez et al., 2021; Lopes et al., 2020; Ward et al., 2018, 2020; Xie et al., 2021). In addition, since IL-15 shares IL-2Rβ and γ_c_ with the IL-2 receptor but has a distinct α chain (Waldmann, 2006), IL-15 therapies have been designed that preferentially target Effs over IL-2Rα^High^ Tregs (Knudson et al., 2020), for example, the current clinical candidate ALT-803 (Romee et al., 2018; Rubinstein et al., 2006; Wrangle et al., 2018; Xu et al., 2013).

Another approach, pioneered by Boyman et al. (2006), built on seminal work (Finkelman et al., 1993) in developing cytokine/antibody complexes to combine IL-2 with anti-IL-2 antibodies that bias cytokine activity. These complexes increase therapeutic efficacy and reduce toxicity of the cytokine by extending its *in vivo* half-life (Roopenian and Akilesh, 2007) and selectively targeting its functions toward particular immune cell subsets (Arenas-Ramirez et al., 2016; Boyman et al., 2006; De Paula et al., 2020; Karakus et al., 2020; Lee et al., 2020b; Spangler et al., 2015a; Tomala et al., 2009; Trotta et al., 2018; Yokoyama et al., 2018). Trotta et al. (2018) discovered and mechanistically described a human antibody (F5111) against hIL-2 that biases its activities toward Tregs. F5111 sterically blocks hIL-2 binding to IL-2Rβ and also allosterically reduces hIL-2 affinity for IL-2Rα. Receptor activation is gated by hIL-2/antibody dissociation, and interaction of the hIL-2/F5111 complex with IL-2Rα destabilizes cytokine/antibody interactions, leading to selective IL-2Rα^High^ Treg activation. This paradigm resembles the exchange/release mechanism observed for the anti-mouse IL-2 (mIL-2) antibody JES6–1 and the anti-hIL-2 antibody UFKA-20, both of which bias IL-2 toward Tregs (Boyman et al., 2006; Karakus et al., 2020; Spangler et al., 2015a). An affinity matured version of F5111 (F5111.2) preferentially expanded Tregs and ameliorated autoimmune diseases in mice (Trotta et al., 2018). However, clinical translation of cytokine/antibody complexes is complicated by the need for dosing ratio optimization and by IL-2 dissociation, which leads to off-target effects. Moreover, use of a single agent rather than a multi-component mixture facilitates the clinical approval pathway. For instance, investigational new drug-enabling nonhuman primate studies of cytokine/antibody complexes require use of the matched species cytokine, but the antibody may not cross-react with nonhuman primate cytokines.

To overcome the limitations of IL-2/antibody complexes, the cytokine has been genetically fused to anti-IL-2 antibodies (Sahin et al., 2020; Spangler et al., 2018; Tomala et al., 2013). In one example, a single-chain fusion protein (immunocytokine [IC]) comprising mIL-2 and the JES6–1 antibody led to superior autoimmune disease control in mice compared with the mIL-2/JES6–1 complex (Spangler et al., 2018). Here, we designed an IC linking hIL-2 to the F5111 antibody (termed F5111 IC) that preferentially promotes mouse and human Treg activation. By modulating the cytokine/antibody affinity, we developed IC variants with a range of immune activation potencies. The parent F5111 IC was poised at an affinity optimum and showed greater Treg bias than hIL-2/antibody complexes. Finally, we established the therapeutic promise of F5111 IC in mouse models of colitis and immune checkpoint inhibitor-induced diabetes mellitus.

## RESULTS

### Immunocytokine design and optimization

To advance development of F5111 IC, we first produced the F5111 human immunoglobulin G1 (IgG1) antibody. Binding studies using the yeast surface display platform (Boder and Wittrup, 1997) revealed that recombinantly expressed F5111 bound yeast-displayed hIL-2 with the expected affinity (Trotta et al., 2018), and the antibody did not cross-react with mIL-2 (Figure S1A).

**Figure 1.**
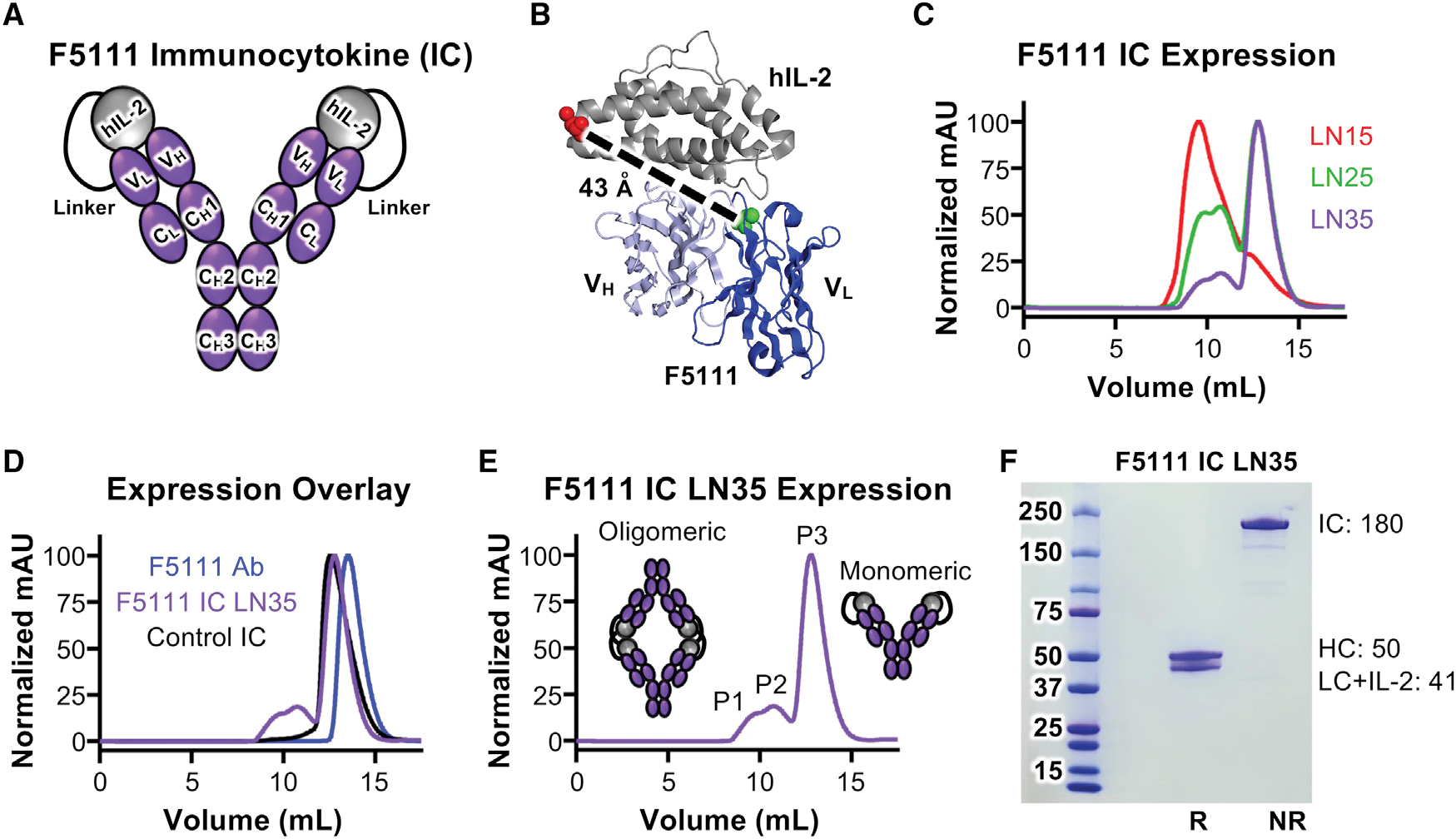
Design and production of F5111 IC (A) Schematic of F5111 IC design. (B) Structure of the hIL-2/F5111 complex (PDB: 5UTZ), showing the distance between the C terminus of hIL-2 (red) and the N terminus of the F5111 antibody LC (green). (C) SEC trace overlay of F5111 ICs. (D) SEC trace overlay of F5111 antibody (Ab) and ICs. (E) SEC trace of F5111 IC LN35 with peaks (P1, P2, and P3) denoted. (F) SDS-PAGE analysis of F5111 IC LN35 P3. R, reducing; NR, non-reducing. Molecular weights (kDa) are indicated on the right. See also Figure S1 and Table S1.

F5111 IC was constructed by tethering hIL-2 to the N terminus of the light chain (LC) of the full-length human F5111 antibody with a flexible linker (Figure 1A). The mechanism of action for biased immune activation by hIL-2/antibody complexes requires cytokine dissociation, and we hypothesized that fusing the cytokine to the antibody could hinder this dissociation through enhanced avidity effects (Spangler et al., 2018). Thus, to reduce the intramolecular cytokine/antibody affinity and enhance hIL-2 release, we formulated the IC using F5111 rather than the affinity matured F5111.2. The hIL-2/F5111 complex structure (Trotta et al., 2018) shows that the F5111 LC N terminus is 43 Å from the hIL-2 C terminus (Figure 1B); therefore our initial design (denoted F5111 IC LN15) used a 15 amino acid (Gly_4_Ser)_3_ linker to enable intramolecular engagement. Constructs with 25 amino acid (Gly_4_Ser)_5_ and 35 amino acid (Gly_4_Ser)_7_ linkers were also designed (F5111 IC LN25 and F5111 IC LN35, respectively). Control IC was constructed by replacing the variable chains of the F5111 IC LN35 construct with those of an irrelevant antibody (Honegger et al., 2005) (Table S1).

ICs were produced in mammalian cells and purified via protein G affinity chromatography and size-exclusion chromatography (SEC). Compared with the F5111 antibody, which eluted as a single monodisperse peak, F5111 IC LN15 eluted earlier and showed two broader peaks (Figures 1C and 1D), suggesting possible oligomerization of F5111 IC LN15. Analysis of F5111 IC LN25 and LN35 revealed three peaks (P1, P2, and P3) (Figures 1C–1E). Elution volumes suggested that P1 and P2 contained oligomeric structures, whereas P3 contained monomeric IC (Figures 1D and 1E). Higher-order oligomers likely indicate binding of linked hIL-2 to F5111 on a neighboring IC (intermolecular assembly) rather than intramolecular assembly (Figure 1E), presumably due to linker constraint of intramolecular interaction between hIL-2 and F5111. Indeed, we saw less oligomerization as we increased linker length. Control IC (which does not assemble intramolecularly or intermolecularly) eluted as a single peak that coincided with P3 of F5111 IC LN25 and LN35, further suggesting that P3 represents monomeric IC. SDS-PAGE verified IC purity (Figure 1F). Henceforth, F5111 IC LN25 and F5111 IC LN35 refer to P3 unless otherwise specified. Also, F5111 IC LN15 indicates the majority peak (Figure 1C).

### F5111 ICs are intramolecularly assembled and exhibit expected binding properties

**Figure 2.**
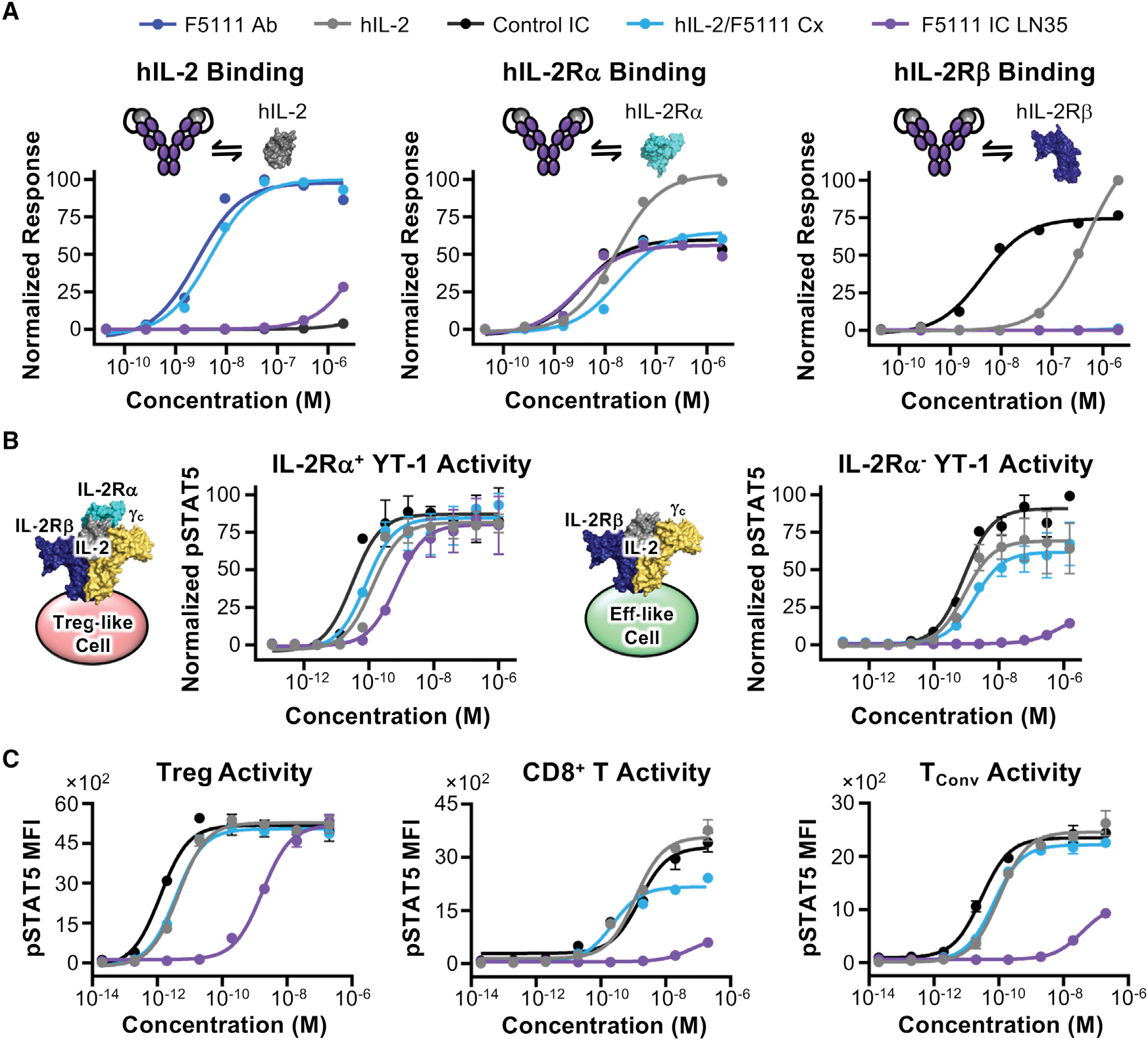
F5111 IC blocks IL-2 binding to IL-2Rβ and biases toward Treg activation (A) Equilibrium biolayer interferometry-based titrations of F5111 antibody (Ab), hIL-2, control IC, hIL-2/F5111 complex (Cx, 1:1 molar ratio), and F5111 IC LN35 binding to hIL-2 (left), hIL-2Rα (middle), or hIL-2Rβ (right). (B) STAT5 phosphorylation response of IL-2Rα^+^ (left) and IL-2Rα^−^ (right) YT-1 cells stimulated with hIL-2, control IC, hIL-2/F5111 Cx (1:1 molar ratio), or F5111 IC LN35. (C) STAT5 phosphorylation responses of stimulated human Treg (left), CD8^+^ T (middle), and T_Conv_ (right) cells. Data represent mean ± SD (n = 3). See also Figures S1 and S2; Tables S2, S3, and S4.

To confirm proper assembly and functionality of F5111 ICs, we measured binding to hIL-2, hIL-2Rα, and hIL-2Rβ using bio-layer interferometry. If properly assembled, F5111 IC would not engage hIL-2 since the hIL-2 within the IC is bound to the antibody. F5111 antibody bound to hIL-2 as expected (Trotta et al., 2018), whereas F5111 IC LN15, F5111 IC LN25, and F5111 IC LN35 showed impaired hIL-2 binding (Figures 2A, left, S1C, left, and S1D, left; Table S2), reflecting intramolecular assembly of the ICs. Trace hIL-2 binding to ICs suggested transient exchange between intramolecular and intermolecular interactions. hIL-2 binding decreased as linker length increased, reinforcing that longer linker lengths enhance IC assembly. The three peaks that eluted from F5111 IC LN25 showed similarly impaired hIL-2 binding (Figure S1E, left). As expected, binding was observed between hIL-2 and the hIL-2/F5111 complex (1:1 molar ratio) due to unoccupied sites on F5111 (Figure 2A, left).

We expected F5111 IC to interact with hIL-2Rα since the receptor-binding epitope on hIL-2 is not directly obstructed by the antibody. F5111 ICs with varying linker lengths bound hIL-2Rα with similar affinities, all of which were >2-fold higher than those of free hIL-2 and hIL-2/F5111 complex due to bivalent cytokine presentation (Figures 2A, middle, S1C, middle, and S1D, middle; Table S2). Control IC had a 5-fold higher affinity for hIL-2Rα compared with free IL-2 (Figure 2A, middle; Table S2). A 2-fold lower hIL-2Rα affinity was observed for F5111 IC LN15 compared with F5111 IC LN25 and F5111 IC LN35, likely due to oligomerization. Indeed, among the three peaks of F5111 IC LN25, P3 had the highest and P1 had the lowest IL-2Rα affinity (Figure S1E, middle).

In contrast with hIL-2Rα, hIL-2Rβ should *not* bind F5111 IC since F5111 fully blocks the receptor binding epitope on hIL-2. Control IC had a 100-fold hIL-2Rβ affinity improvement over free hIL-2 due to its bivalency (Figure 2A, right; Table S2). Whereas binding to hIL-2Rβ was completely abolished for hIL-2/F5111 complex (Figure 2A, right; Table S2), F5111 IC LN15 showed similar hIL-2Rβ affinity to free hIL-2 (Figure S1C, right), indicating deficient intramolecular assembly. Lengthening the linker eliminated these issues, as F5111 IC LN25 and F5111 IC LN35 did not engage hIL-2Rβ (Figures 2A, right, S1C, right, and S1D, right; Table S2). F5111 IC LN25 P1 and F5111 IC LN25 P2 bound hIL-2Rβ weakly, whereas F5111 LN25 P3 did not bind hIL-2Rβ (Figure S1E, right). Overall, hIL-2 cytokine and receptor binding studies confirmed proper assembly and function of F5111 ICs.

### F5111 ICs demonstrate Treg bias *in vitro*

IL-2-dependent STAT5 phosphorylation (pSTAT5) was assessed on YT-1 human NK cells that either express (Treg-like) or lack (Eff-like) IL-2Rα (Kuziel et al., 1993). ICs were compared with hIL-2/F5111 complex (1:1 molar ratio), and increasing antibody-to-cytokine ratio did not affect signaling potency (Figure S1B; Table S3).

On IL-2Rα^+^ (Treg-like) cells, F5111 IC LN15, F5111 IC LN25, and F5111 IC LN35 induced robust activation, similar to free hIL-2 and hIL-2/F5111 complex (Figures 2B, left, and S1F; Table S3). Control IC was more potent than free hIL-2 due to its bivalency, whereas F5111 ICs were slightly less potent due to incomplete cytokine/antibody dissociation. On IL-2Rα^−^ (Eff-like) cells, F5111 IC LN15 was 15-fold less potent than free hIL-2 and control IC, and F5111 IC LN25 and F5111 IC LN35 induced little to no activation (Figures 2B, right, and S1G; Table S3). In contrast, hIL-2/F5111 complex was only ~2-fold less potent than free hIL-2 on IL-2Rα^−^ cells, highlighting the enhanced IL-2Rα^+^ cell bias of ICs versus the complex. The two peaks of F5111 IC LN15 elicited ~10-fold weaker activation of IL-2Rα^−^ cells relative to free hIL-2 (Figure S1H, top; Table S3). For F5111 IC LN25, P1 and P2 EC_50_ values were 50- and 100-fold weaker than that of hIL-2, respectively, whereas P3 was >2,000-fold weaker (Figure S1H, bottom; Table S3). All three peaks of F5111 IC LN25 were less active on IL-2Rα^−^ cells compared with F5111 IC LN15, confirming that increased linker length improved IC assembly. The potencies of F5111 IC LN25 and F5111 IC LN35 on IL-2Rα^+^ and IL-2Rα^−^ cells were similar; however, less oligomerization was observed for F5111 IC LN35 (Figure 1B). Thus, we proceeded with F5111 IC LN35, hereafter denoted F5111 IC.

To confirm Treg bias in a mixed cell population, IC activity was interrogated on human peripheral blood mononuclear cells (PBMCs). F5111 IC was less potent than free hIL-2 and IL-2/F5111 complex (~500-fold) as well as control IC (~1,000-fold) on Tregs due to incomplete cytokine/antibody dissociation (Figure 2C, left; Table S4). However, equivalent E_Max_ values were achieved for all constructs. F5111 IC did not activate on CD8^+^ T cells, as indicated by reduced potency and ~80% reduction in E_Max_ value compared with free hIL-2 and control IC (Figure 2C, middle; Table S4). In contrast, hIL-2/F5111 complex showed improved potency on CD8^+^ T cells with a milder 35% reduction in E_Max_ relative to free IL-2 and control IC. Similarly, F5111 IC induced little to no activation of conventional CD4^+^ T cells (T_Conv_), with 55% lower E_Max_ compared with free hIL-2 and control IC (Figure 2C, right; Table S4). However, hIL-2/F5111 complex activated T_Conv_ cells with similar potency and E_Max_ as free IL-2, presumably due to complex dissociation. Control IC was more potent than free hIL-2 due to bivalency. Collectively, PBMC studies revealed that F5111 IC, but not IL-2/F5111 complex, is strongly biased toward Treg versus Eff activation due to preferential engagement of IL-2Rα^High^ cell subsets.

To understand the more dramatic potency reduction for F5111 IC compared with free hIL-2 on human Tregs compared with IL-2Rα^+^ YT-1 cells, we quantified IL-2Rα expression on these cells. IL-2Rα mean fluorescence intensity (MFI) on Tregs was only 40-fold greater than the fluorescence minus one (FMO) control, whereas IL-2Rα MFI was 75-fold greater than FMO control for IL-2Rα^+^ YT-1 cells (Figure S2B, left), indicating that YT-1 cells express more IL-2Rα than Tregs. IL-2Rα MFI levels on CD8^+^ T, T_Conv_, and IL-2Rα^−^ YT-1 cells were close to FMO background (Figure S2B, right). As F5111 IC binding requires IL-2Rα-dependent disruption of the cytokine/antibody interaction, lower expression of IL-2Rα on human primary cells rationalizes the weakened activity of F5111 IC on Tregs.

### Tuning IC intramolecular affinity modulates IL-2 receptor binding and Treg bias

**Figure 3.**
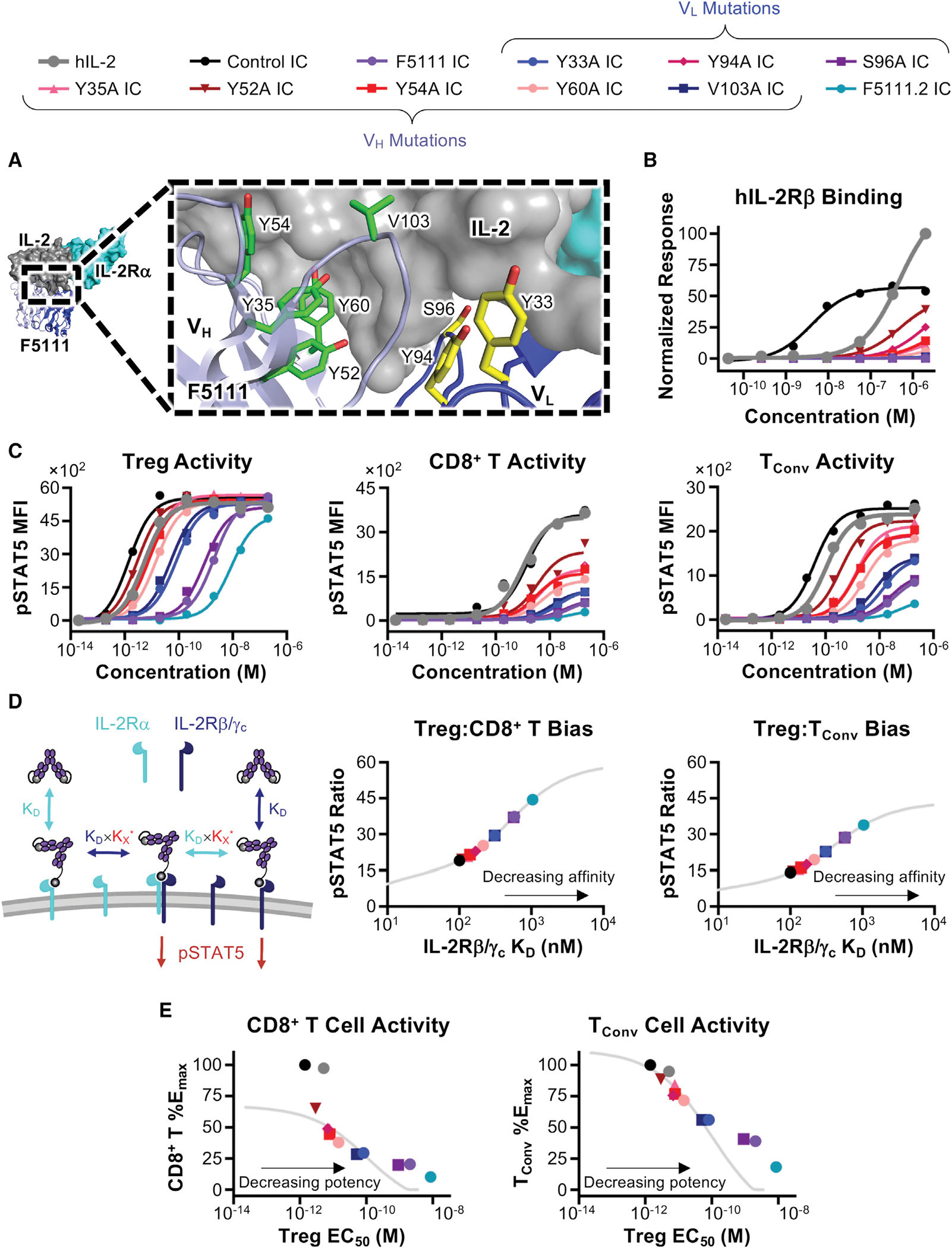
Tuning IC intramolecular affinity modulates Treg bias (A) hIL-2/F5111 crystal structure (PDB: 5UTZ) with alanine-mutated residues shown in yellow (LC) or green (HC). Human IL-2Rα is overlaid from the IL-2 quaternary complex structure (PDB: 2B5I). (B) Equilibrium biolayer interferometry-based titrations against hIL-2Rβ. (C) STAT5 phosphorylation responses of stimulated human Treg (left), CD8^+^ T (middle), and T_Conv_ (right) cells. (D) Schematic of multivalent binding model (left). Predicted Treg:CD8^+^ T (middle) and Treg:T_Conv_ (right) pSTAT5 ratios for ICs at 10 pM concentration are plotted against predicted IL2Rβ/γ_c_ K_D_ (nM). (E) Predicted (lines) and experimental (points) percent control IC pSTAT5 E_Max_ on CD8^+^ T (left) or T_Conv_ (right) cells plotted against the predicted (lines) or experimental (points) pSTAT5 EC_50_ on Tregs for each IC. See also Figure S3; Tables S2 and S4.

Based on our mechanistic understanding of F5111 IC activity, we hypothesized that modulating cytokine/antibody affinity would impact relative engagement of IL-2Rα^High^ versus IL-2Rα^Low^ cells, enabling optimization of IC bias on different cell types with various IL-2Rα expression levels. We conjectured that reducing the IL-2/F5111 interaction affinity would lead to enhanced IL-2 signaling, particularly on human Tregs. Informed by the structure of the IL-2/F5111 complex (Trotta et al., 2018), we rationally designed a panel of eight single-point alanine mutations of the F5111 antibody, including three variable LC (V_L_) (Y33, Y94, and S96) and five variable HC (V_H_) (Y35, Y52, Y54, Y60, and V103) residues at the cytokine/antibody interface (Figure 3A). Each F5111 variant and the affinity matured F5111.2 antibody (Rondon et al., 2015) were produced as ICs with 35 amino acid linkers (Figure S3A).

Bio-layer interferometry studies showed that F5111 IC variants minimally engaged hIL-2, confirming their intramolecular assembly (Figure S3B; Table S2). All IC variants bound hIL-2Rα with similar affinities, comparable with the parent F5111 IC (Figure S3B; Table S2). All IC variants showed significantly weaker IL-2Rβ binding compared with free hIL-2 and control IC, indicating successful receptor blockade (Figure 3B; Table S2). However, whereas most IC variants completely ablated hIL-2Rβ binding, IC variants Y94A, Y35A, Y52A, Y54A, and Y60A detectably bound hIL-2Rβ at high concentrations, suggesting weaker cytokine/antibody interactions for these clones.

We wondered whether hIL-2Rβ binding differences between IC variants would impact IL-2 signaling bias. In human PBMC studies, all F5111 IC variants either maintained or enhanced Treg potency compared with the parent F5111 antibody (Figure 3C, left; Table S4), indicating that the mutations weakened cytokine/antibody binding, as intended. IC variants were grouped into three cohorts: the “high”-potency group (Y94A, Y35A, Y52A,Y54A, and Y60AICs), which were of similar potency to control IC; the “intermediate”-potency group (Y33A and V103A ICs), which were ~50-fold weaker than control IC; and the “low”-potency group (parent F5111 IC and the S96A IC), which were ~1,000-fold weaker than control IC. F5111.2 IC impaired the Tregpotency (>6,000-fold weaker thancontrol IC) due to reduced cytokine/antibody dissociation. As anticipated, weakened cytokine/antibody affinityalso potentiated the activity of F5111IC variants on both CD8^+^ T cells (Figure 3C, middle; Table S4) and T_Conv_ cells (Figure 3C, right; Table S4). However, all IC variants showed impaired Eff activation compared with free hIL-2 and control IC, meaning they retained some Treg bias. IC variants with the highest Treg potencies led to the most potent CD8^+^ T and T_Conv_ cell activation, and F5111.2 IC, which was the least potent on Tregs, elicited the weakest Eff activation. Moreover, signaling potency on both Treg and Effs was directly correlated with extent of hIL-2Rβ engagement, as anticipated.

F5111 IC variants represent a panel of IL-2Rα “probes,” as illustrated by IL-2Rα MFI analysis within the activated cell populations for various immune cell subsets. At saturating concentrations, the mean IL-2Rα MFIs of activated Tregs were similar following treatment with all F5111 ICs except for the significantly less potent F5111.2 IC (Figure S3C, left). This finding suggests that Treg cell IL-2Rα expression is sufficient to induce cytokine/antibody dissociation for all F5111 ICs other than F5111.2 IC, which requires more IL-2Rα to induce cytokine/antibody dissociation. On CD8^+^ T and T_Conv_ cells, ICs that induced more potent activation required lower levels of IL-2Rα to stimulate IL-2 signaling (Figure S3C, middle and right).

To further explore the relationship between cytokine/antibody affinity and Treg bias of ICs we developed a multivalent binding model that predicts F5111 IC signaling properties in specific immune cell subsets based on receptor affinity (Figure 3D, left). This model accurately predicted activities of the IC variants (Figures S3D–S3F) and inferred hIL-2Rβ/γ_c_ affinities (Figures S3G and S3H), which we could not measure experimentally since IC variants did not reach binding saturation. We plotted the predicted affinity of each IC against the predicted Treg:CD8^+^ T and Treg:T_Conv_ pSTAT5 MFI ratios at a fixed concentration (Figure 3D, middle and right). As IC affinity toward hIL-2Rβ/γ_c_ decreased, Treg bias increased. Bias resulted from attenuated signaling on both CD8^+^ T and T_Conv_ cells but was also accompanied by decreased activation of Tregs (Figures 3C and 3E), illustrating the trade-off between Treg activation and selectivity. We selected three IC variants for further analysis that improved Treg potency while minimizing Eff activation (Y60A, Y33A, and V103A ICs).

To improve their therapeutic potential, we introduced the N297A mutation into the Fc region of all ICs. This mutation prevents glycosylation, which significantly impairs Fc γ receptor binding and thus reduces antibody effector functions (Delidakis et al., 2022; Mimura et al., 2000, 2001; Saunders, 2019; Tao et al., 1993; Wang et al., 2018). Bio-layer interferometry studies showed no differences in hIL-2, hIL-2Rα, and hIL-2Rβ binding between F5111 IC with and without the N297A mutation (Figure S4A; Table S2). Human PBMC signaling assays confirmed that N297A mutation did not affect the activity of control IC, F5111 IC, or IC variants (Figures S4B and S4C; Table S4). Thus, IC will hereafter denote the IC with the N297A mutation unless otherwise indicated.

### Parent F5111 IC maximizes Treg expansion bias

We sought to determine whether the *in vitro* IL-2 signaling bias of our engineered ICs toward activation of Tregs would translate into selective expansion of Tregs in mice. We first evaluated IC potencies on splenocytes isolated from non-obese diabetic (NOD) mice. IC activation trends on mouse primary cells were similar to those on human PBMCs (Figures S4C and S4D; Tables S4 and S5), albeit with weaker potency due to the lower affinity of hIL-2 for mIL-2 versus hIL-2 receptors (Spangler et al., 2015a).

To inform *in vivo* dosing, we plotted *in vitro* Treg:CD8^+^ T and Treg:T_Conv_ pSTAT5 MFI ratios as a function of IC concentration (Figure S4E). Each IC variant exhibited a unique optimum concentration and maximum level of Treg bias, and variants that were more potent on Tregs had lower optimum concentrations and higher magnitude biases.

**Figure 4.**
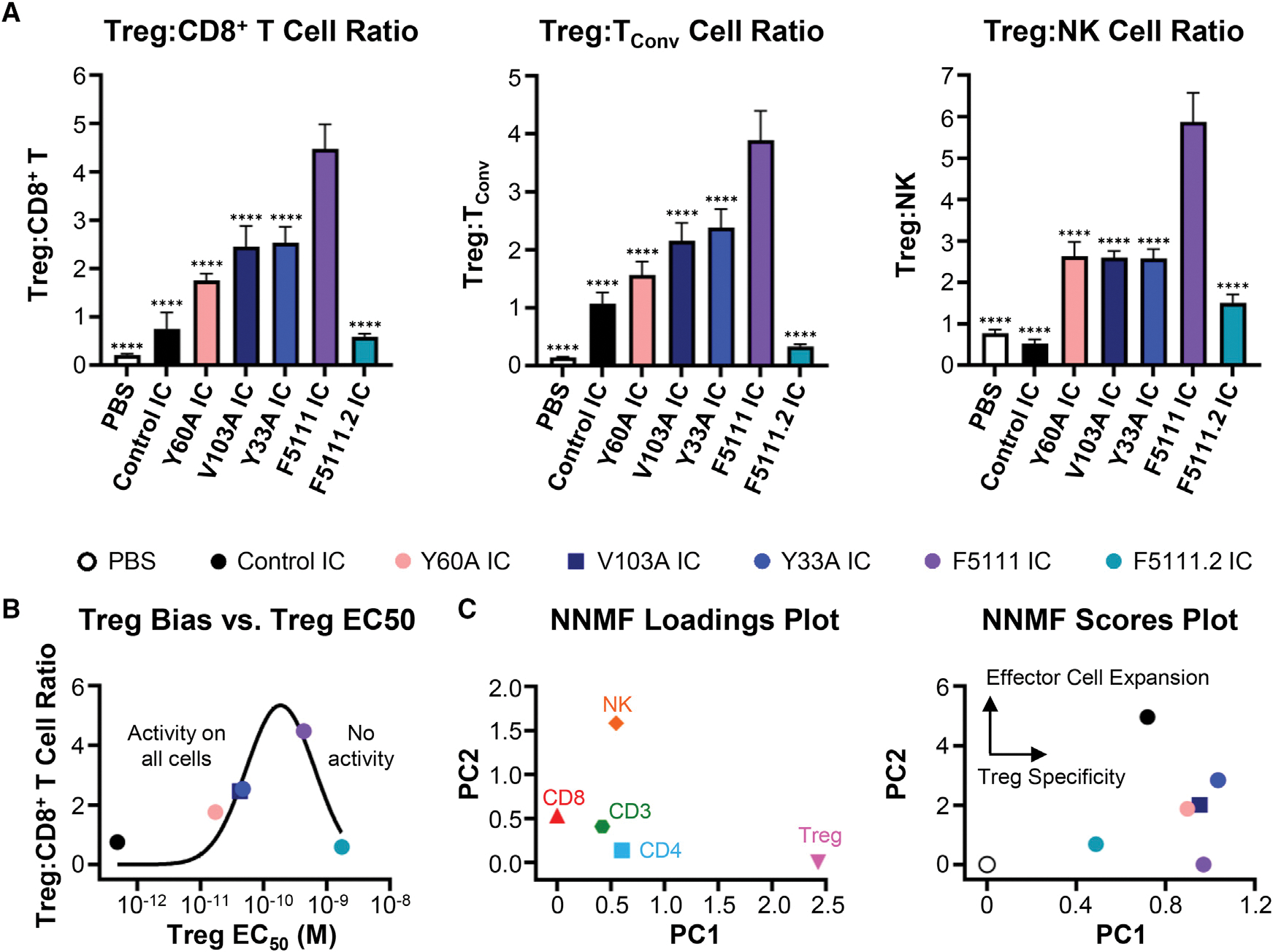
Parent F5111 IC induces maximum Treg expansion bias (A) Treg:CD8^+^ T (left), Treg:T_Conv_ (middle), and Treg:NK (right) cell ratios in NOD mice spleens after four daily treatments with PBS (n = 4) or 8.2 μg (1.5 μg IL-2 equivalence) control IC (n = 4), Y60A IC (n = 5), V103A IC (n = 4), Y33A IC (n = 4), F5111 IC (n = 5), or F5111.2 IC (n = 4). Data represent mean + SD. Statistical significance compared with F5111 IC is shown (all data in Table S6). (B) Plot of Treg:CD8^+^ T cell *in vivo* expansion ratio versus pSTAT5 EC_50_ on human Tregs (Figure S4C, left; Table S4). Data were fit to a Gaussian distribution. (C) Non-negative matrix factorization (NNMF) loadings plot (left) and scores plot (right) of *in vivo* cell subset expansion studies in NOD mice. *p ≤ 0.05, **p ≤ 0.01,</p>***p ≤ 0.001, ****p ≤ 0.0001. See also Figures S4 and S5.

NOD mice were treated with each IC and immune cell subset expansion was evaluated in harvested spleens (Figure S5A). Y33A IC, V103A IC, and Y60A IC showed significant Treg bias (Figure 4A), but remarkably, the parent F5111 IC led to the most biased Treg expansion among the ICs tested, indicating that the optimum hIL-2/antibody affinity for Treg bias corresponded to that of the parent F5111 IC. This is visualized by plotting the Treg:CD8^+^ T cell ratio against Treg potency for each IC (Figure 4B; Table S4). If Treg activity is weak (as for F5111.2 IC), no activity is observed on any immune cell subset. If the Treg activity is too potent (as for control IC), all immune cell subsets are activated, confounding Treg bias. The optimum Treg EC_50_ lies between these extrema, and F5111 IC is close to this optimum. Non-negative matrix factorization was performed using a two-component analysis, revealing that F5111 IC had equivalent Treg specificity IC variants while maintaining inhibition of Eff activation (Figure 4C).

We also evaluated our panel of IC variants in a humanized mouse model. BALB/c Rag2^−/−^γ_c_^−/−^ H2^d^ mice engrafted with human PBMC were treated with ICs and immune cell subset expansion in the peritoneum was measured. In contrast to NOD mouse results, optimal Treg expansion bias was observed for Y60A IC and Y33A IC (Figure S4F), which have weaker cytokine/antibody affinity than the parent F5111 IC. Thus, the optimal Treg-promoting therapy may vary between mice and humans.

To assess the impact of Fc effector function on IC-induced Treg expansion, we compared immune activation by the IC with and without the N297A mutation. F5111 IC with the N297A mutation (impaired Fc effector function) led to significantly more Treg bias and >3-fold more Treg expansion (Figure S5B); thus we elected to employ F5111 IC with impaired Fc effector function in mouse models of disease.

### F5111 IC exhibits greater Treg bias than hIL-2/F5111.2 complex

We hypothesized that the IC could have stability improvements over cytokine/antibody complexes; thus, we compared the previously reported lead IL-2/antibody complex (hIL-2/F5111.2 complex) (Trotta et al., 2018) to our lead construct (F5111 IC). The N297A mutation was installed in the F5111.2 antibody for consistency with F5111 IC. Bio-layer interferometry studies showed that the F5111.2 antibody and hIL-2/F5111.2 complex (1:1 molar ratio) bound to hIL-2, whereas F5111 IC and control IC had little to no hIL-2 binding (Figure S5C, left; Table S2). hIL-2/F5111.2 complex bound hIL-2Rα with ~18-fold weaker affinity than F5111 IC and control IC and ~3-fold weaker affinity than free hIL-2 (Figure S5C, middle; Table S2), consistent with previous data (Trotta et al., 2018). Both hIL-2/F5111.2 complex and F5111 IC fully blocked binding to hIL-2Rβ (Figure S5C, right; Table S2).

**Figure 5.**
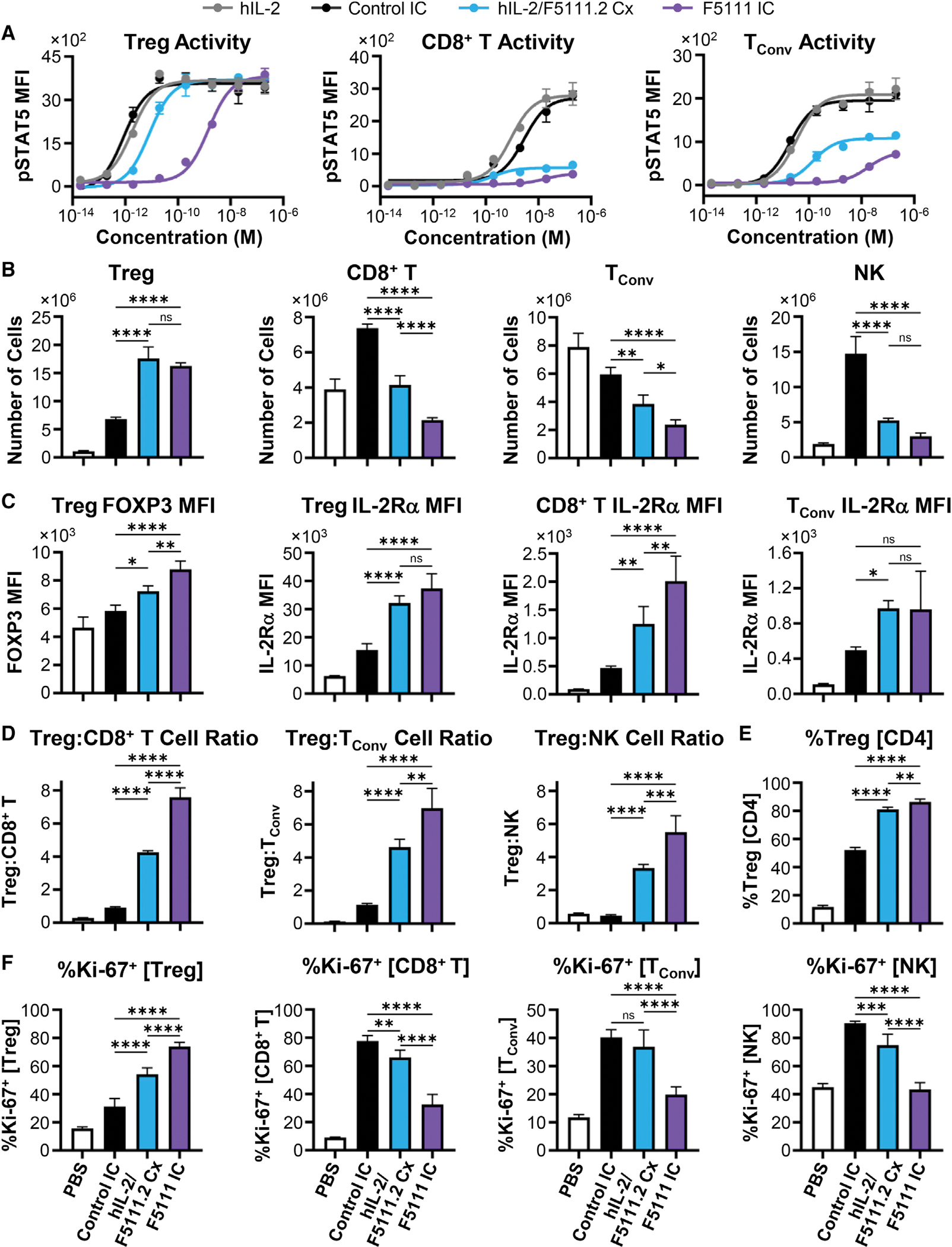
F5111 IC shows greater Treg bias than hIL-2/F5111.2 complex (A) pSTAT5 response of stimulated human Treg (left), CD8^+^ T (middle), and T_Conv_ (right) cells. hIL-2/F5111.2 complex (Cx) was at a 1:1 molar ratio. (B–E) NOD mice (n = 4 per group) were treated daily for 4 days with PBS, 1.5 μg hIL-2 with 6.6 μg F5111.2 antibody (1:2 molar ratio), or 8.2 μg (1.5 μg IL-2 equivalence) control IC or F5111 IC. (B) Number of Treg, CD8^+^ T, T_Conv_, and NK cells. (C) MFI of FOXP3 within Tregs (left) and IL-2Rα MFI within Treg, CD8^+^ T, and T_Conv_ cells. (D) Treg:CD8^+^ T (left), Treg:T_Conv_ (middle), and Treg:NK cell (right) ratios. (E) Percent Tregs within the CD4^+^ T cell population. (F) Percent Ki-67^+^ cells within Treg, CD8^+^ T, T_Conv_, and NK cells from NOD mice (n = 5 per group) treated as in (B–E). Data represent mean ± SD. Statistical significance between control IC, hIL-2/F5111.2 Cx, and F5111 IC shown. *p ≤ 0.05, **p ≤ 0.01, ***p ≤ 0.001, ****p ≤ 0.0001. See also Figure S5; Table S4.

On human Tregs, F5111 IC and hIL-2/F5111.2 complex (1:1 molar ratio) were ~1,700- and ~10-fold weaker than control IC on Tregs, respectively (Figure 5A, left; Table S4). On CD8^+^ T cells, F5111 IC and hIL-2/F5111.2 reduced the E_Max_ by 85% and 80%, respectively, relative to control IC (Figure 5A, middle; Table S4). On T_Conv_ cells, F5111 IC was ~1,000-fold weaker with a 60% reduction in E_Max_, whereas hIL-2/F5111.2 complex was ~7-fold weaker with a 50% reduction in E_Max_ relative to control IC (Figure 5A, middle; Table S4).

NOD mouse studies showed that both F5111 IC and hIL-2/F5111.2 complex (2:1 cytokine:antibody molar ratio) led to 15-fold increases in the total number of Tregs compared with untreated mice, and induced 3-fold more Treg expansion than control IC (Figure 5B). F5111 IC and hIL-2/F111.2 complex treatment also led to significantly less CD8^+^ T, T_Conv_, and NK cell expansion compared with control IC, with the IC inducing less Eff expansion than the complex (Figure 5B). Both F5111 IC and hIL-2/F5111.2 complex elevated expression of forkhead box P3 (FOXP3) within the Treg population compared with control IC, with the IC inducing significantly more FOXP3 expression than the complex (Figure 5C). In addition, F5111 IC and hIL-2/F5111.2 complex increased IL-2Rα expression on Treg, CD8^+^ T, and T_Conv_ cells relative to control IC (Figure 5C). Both F5111 IC and hIL-2/F5111.2 complex augmented the Treg:CD8^+^ T, Treg:T_Conv_, and Treg:NK cell ratios compared with control IC, with the IC eliciting significantly more bias than the complex (Figure 5D). Notably, F5111 IC treatment resulted in >80% Tregs within the CD4^+^ population, a massive expansion over the 10% Treg proportion observed in saline-treated mice (Figure 5E). F5111 IC treatment also significantly increased the percentage of Ki-67^+^ (proliferating) Tregs and lowered the percentage of Ki-67^+^ CD8^+^ T, T_Conv_, and NK cells compared with hIL-2/F5111.2 complex and control IC (Figure 5F). Taken together, our *in vivo* data showcase the Treg-biasing capacity of F5111 IC, and demonstrate its superiority to cytokine/antibody complex.

### F5111 IC induces durable expansion of functional Tregs

**Figure 6.**
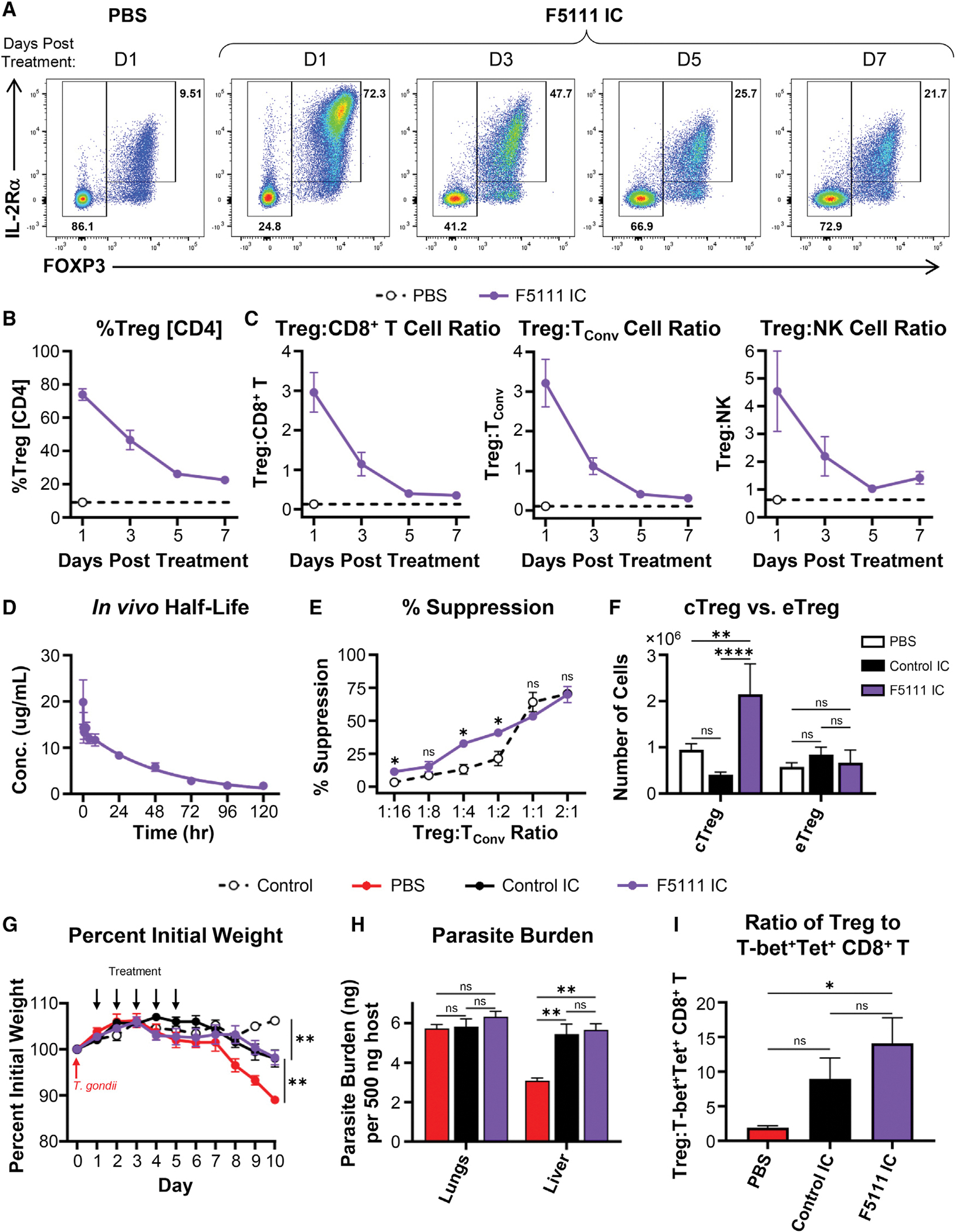
F5111 IC expands functional Tregs without compromising infection immunity (A–C) C57BL/6 mice (n = 3 per group) were treated daily for 4 days (intraperitoneally) with PBS or 8.2 μg F5111 IC (1.5 μg IL-2 equivalence) and spleens were harvested at the indicated times. (A) Representative flow plots showing Treg percentage and IL-2Rα versus FOXP3 levels in CD4^+^ T cells at the indicated times. (B) Percent Tregs within CD4^+^ T cells at the indicated times. (C) Treg:CD8^+^ T (left), Treg:T_Conv_ (middle), and Treg:NK (right) at the indicated times. Dashed lines show baseline values for the PBS-treated cohort harvested 1 day after the last dose. (D) Serum half-life of F5111 IC in C57BL/6 mice (n = 5) treated retro-orbitally with 2 mg/kg F5111 IC (~0.4 mg/kg IL-2 equivalence). (E) C57BL/6 CD45.1 RFP-FOXP3 mice were treated daily for 4 days with PBS (n = 3) or 6.2 μg F5111 IC (1.125 μg IL-2 equivalence, n = 2). Percent proliferation suppression of T_Conv_ cells from untreated C57BL/6 CD45.2 mice (n = 5) by titrating ratios of Tregs from spleens of treated mice is shown. (F) C57BL/6 mice (n = 5 per group) were dosed on days 0, 2, and 4 with PBS or 8.2 μg (1.5 μg IL-2 equivalence) control IC or F5111 IC. Spleens were harvested on day 6. Number of cTregs (CD4^+^FOXP3^+^IL-2Rα^High^BCL-2^High^) and effector (eTregs) (CD4^+^FOXP3^+^IL-2Rα^Low^BCL-2^Low^) are shown. (G–I) C57BL/6 mice were administered 25 cysts of *T. gondii* on day 0. The control group was not given cysts. Starting on day 1, mice were treated daily for 5 days with PBS (control, n = 5; PBS, n = 4) or 8.2 μg (1.5 μg IL-2 equivalence) control IC (n = 5) or F5111 IC (n = 5). Mice were sacrificed on day 10. (G) Mouse weight. (H) Parasite burden. (I) Treg:T-bet^+^Tetramer (Tet)^+^ CD8^+^ T cell ratio in the spleen. (G–I) Mean ± SEM. All other data are mean ± SD. Statistical significance in percent initial weight on day 10 compared with F5111 IC is shown. *p ≤ 0.05, **p ≤ 0.01, ***p ≤ 0.001, ****p ≤ 0.0001. See also Figure S5.

Dose dependence studies of F5111 IC-induced Treg expansion showed that Treg frequency within the CD4^+^ T cell population was consistent down to a dose of 0.5 μg IL-2 equivalence (Figure S5D). Kinetic studies revealed that Treg percentage within the CD4^+^ population was highest 1 day after the final of four daily F5111 IC doses, and the percentage declined monotonically for each subsequent time point but still remained elevated after 1 week (Figures 6A and 6B). Similarly, maximum Treg bias was observed 1 day after the last dose, and bias was mostly gone after 5 days (Figure 6C). Serum levels of F5111 IC in C57BL/6 mice followed a two-phase decay with a fast half-life of ~5 min and a slow half-life of 35 h (Figure 6D).

To characterize the functional properties of Tregs expanded by F5111 IC, we conducted a suppression assay using Tregs isolated from C57BL/6 CD45.1 RFP-FOXP3 mice treated with either saline or F5111 IC. Compared with Tregs isolated from saline-treated mice, Tregs expanded by F5111 IC had equivalent suppressive capacity against naive T_Conv_ cells from C57BL/6 CD45.2 mice. (Figure 6E). We also assessed the phenotype of F5111 IC-expanded Tregs from C57BL/6 mice and found significantly increased numbers of conventional Tregs (cTregs) (CD4^+^FOXP3^+^IL-2Rα^High^BCL-2^High^) but not effector Tregs (CD4^+^FOXP3^+^IL-2Rα^Low^BCL-2^Low^) (Figure 6F). Interestingly, control IC treatment resulted in a reduced number of cTregs compared with saline-treated mice, whereas eTreg numbers were not affected. This aligns with the mechanism of F5111 IC, which biases the molecule toward immune cell subsets with high levels of IL-2Rα.

### F5111 IC treatment does not impair immune response to infection

A potential concern for therapeutic administration of a Treg-promoting agent, such as F5111 IC, is that it may interfere with T cell-mediated clearance of infection (Belkaid and Tarbell, 2009; Belkaid et al., 2006; Oldenhove et al., 2009; Sacks and Anderson, 2004). We therefore probed the effects of F5111 IC in a mouse model of toxoplasmosis. Treatment with either F5111 IC or control IC following *Toxoplasma gondii* infection conferred protection against disease-associated weight loss compared with saline treatment (Figure 6G), and no significant differences were observed in lung parasite burden (Figure 6H). Control IC and F5111 IC led to similar changes in parasite burden in the liver relative to saline treatment. Histological analysis of immune-mediated liver pathology in control IC-treated mice showed exacerbated leukophilia and necrosis compared with saline-treated mice (Figure S5E). Interestingly, despite similar parasite burden to control IC-treated animals, F5111 IC-treated mice showed an overall reduction in immunopathological changes compared with saline-treated mice (Figure S5E). Importantly, F5111 IC and control IC behaved similarly in this model even though F5111 IC significantly increased Treg numbers (Figure S5F) and skewed ratios of Tregs to both T-bet^+^Tetramer^+^ CD8^+^ T cells (Figure 6I) and T-bet^+^Tetramer^+^ CD4^+^ T_Conv_ cells (Figure S5G). Expanded Tregs showed evidence of active proliferation (Figure S5H) and upregulation of IL-2Rα (Figure S5I). Overall, this study suggests that, although F5111 IC promotes Treg expansion, it does not impair infection clearance.

### F5111 IC efficacy in a mouse colitis model

**Figure 7.**
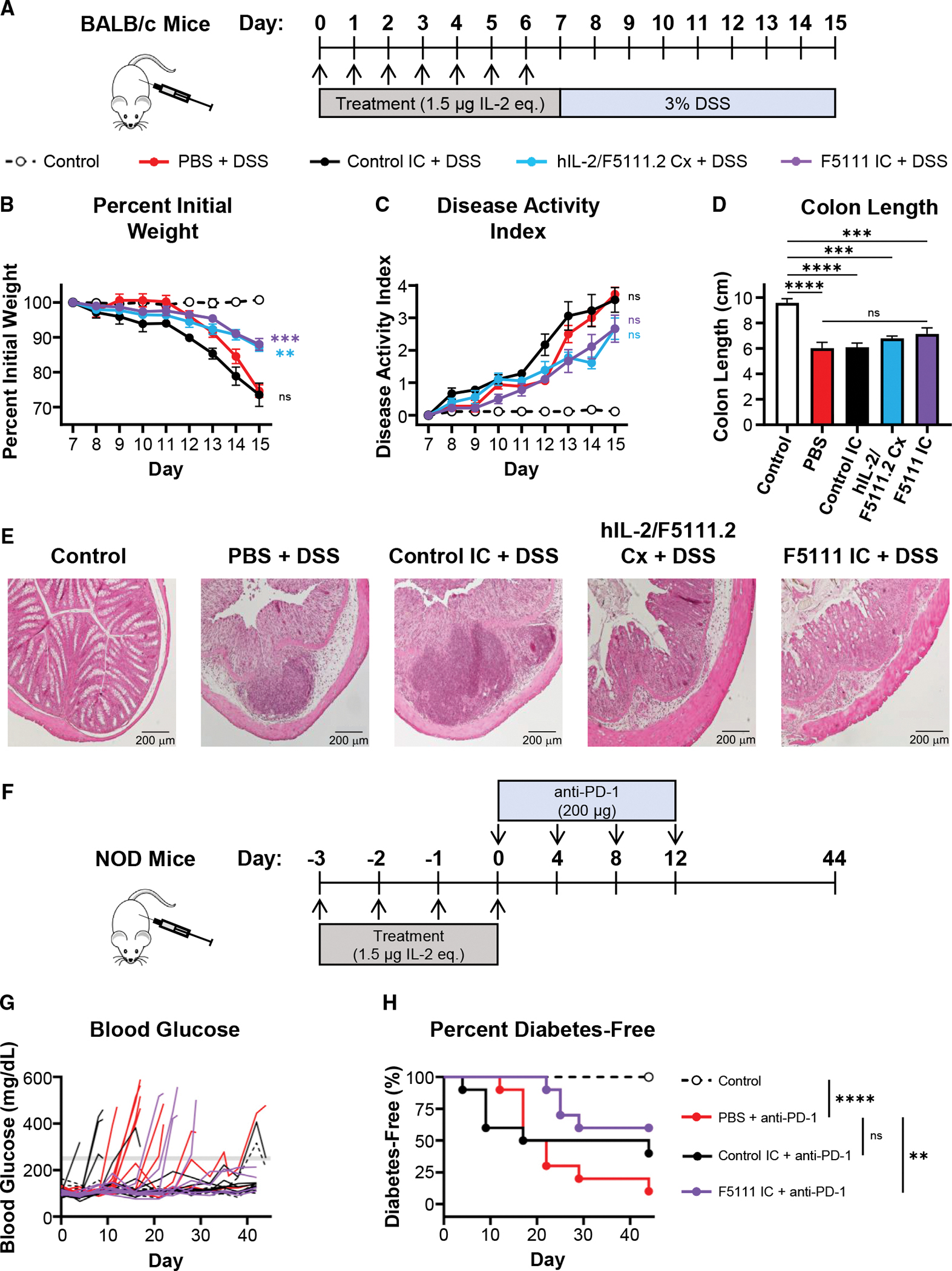
F5111 IC confers protection in mouse models of autoimmune disease (A) BALB/c mice (n = 6 per group) were treated daily for 7 days with PBS (control and PBS), 1.5 μg hIL-2 complexed with 6.6 μg F5111.2 antibody (1:2 molar ratio, hIL-2/F5111.2 Cx), or 8.2 μg (1.5 μg IL-2 equivalence) control IC or F5111 IC. Beginning on day 7, all groups except disease-free control received 3% DSS in their drinking water. (B) Weight change. (C) Disease activity index (DAI). (D) Colon lengths on day 15 (n = 6 PBS, Cx, F5111 IC, control; n = 5 control IC). (E) H&E-stained colons from treated mice (n = 5 control, control IC; n = 6 PBS, Cx, F5111 IC). Scale bar, 200 μm. (B–D) Mean ± SEM. Weight change and DAI plots show significance of control IC-, Cx-, and F5111 IC-treated mice versus PBS-treated mice on day 15. Colon length plot shows significance compared with control group and between PBS- and F5111 IC-treated mice. (F–H) NOD mice (n = 10 per group) were treated with PBS (control and PBS) or 8.2 μg (1.5 μg IL-2 equivalence) control IC or F5111 IC. (G) Blood glucose concentrations. The gray line indicates the 250 mg/dL threshold. (H) Percent diabetes-free mice. Statistical significance compared with mice treated with PBS + anti-PD-1 is shown. *p ≤ 0.05, **p ≤ 0.01, ***p ≤ 0.001, ****p ≤ 0.0001. See also Figure S6.

To evaluate the potential for F5111 IC to prevent autoimmune disease development, we assessed its performance as a prophylactic treatment in a mouse dextran sulfate sodium (DSS)-induced mouse colitis model (Chassaing et al., 2014; Cooper et al., 1993; Okayasu et al., 1990; Spangler et al., 2015a, 2018) (Figure 7A). Both F5111 IC and hIL-2/F5111.2 complex significantly reduced the severity of weight loss and led to decreased disease activity scores on day 15 compared with saline and control IC treatment (Figures 7B, 7C, S6A, and S6B). Relative to control mice without DSS exposure, mice treated with F5111 IC and hIL-2/F5111.2 complex exhibited a less significant colon length reduction compared with mice treated with saline or control IC (Figure 7D). Colon histology analysis revealed that both F5111 IC and hIL-2/F5111.2 complex treatment led to reduced histopathological scores compared with control IC and saline treatment (Figures 7E and S6C). Overall, this colitis model illustrated the capacity of F5111 IC to confer protection against autoimmune disease pathogenesis.

### F5111 IC is protective in a mouse model of immune checkpoint inhibitor-induced diabetes mellitus

To further evaluate the therapeutic potential for F5111 IC, we examined its performance in a mouse model of immune checkpoint inhibitor-induced diabetes mellitus (Ansari et al., 2003; Fife et al., 2006; Hu et al., 2020). This model mimics the etiology of a current clinical concern, in which cancer patients treated with immunotherapies are at risk of developing immune-related adverse events, including diabetes (Quandt et al., 2021; Stamatouli et al., 2018; Young et al., 2018). NOD mice were treated with saline, control IC, or F5111 IC, and then administered anti-mouse PD-1 antibody to induce disease (Figure 7F). Salinetreated mice began developing diabetes on day 12, whereas a proportion of control IC-treated mice showed accelerated diabetes onset starting on day 4 (Figures 7G and 7H). In contrast, all mice treated with F5111 IC remained diabetes free until day 22. On day 44 (before onset of spontaneous disease in the control group that did not receive anti-PD-1), 60% of mice treated with F5111 IC were diabetes free, whereas only 10% of mice in the saline-treated group were diabetes free. Disease-free survival was found to be significantly improved for F5111 IC-treated but not control IC-treated mice compared with salinetreated mice. To further assess the durability of F5111 IC therapy in this model, we performed a similar study over 175 days (Figure S6D). F5111 IC led to significant long-term protection compared with saline in immune checkpoint inhibitor-treated mice, as no additional animals developed diabetes after day 31 (Figures S6E and S6F). Control mice that did not receive anti-PD-1 antibody began developing spontaneous diabetes on day 50 and, by day 157, all mice had disease. These studies highlight that the biased Treg expansion induced by F5111 IC is protective in preventing the onset of immune checkpoint inhibitor-induced diabetes in mice and hint that therapy may also help prevent spontaneous diabetes onset in NOD mice.

## DISCUSSION

Biased cytokines are of great interest as therapeutics due to their potential to harness the power of natural proteins and precisely regulate their biological effects (Spangler et al., 2015b). Ongoing efforts in cytokine targeting include mutein engineering (Peterson et al., 2018; Carmenate et al., 2018; Khoryati et al., 2020; Glassman et al., 2021), selective PEG-ylation (Charych et al., 2016; Dixit et al., 2021; Zhang et al., 2021), and cytokine/antibody complex design (Arenas-Ramirez et al., 2016; Boyman et al., 2006; De Paula et al., 2020; Karakus et al., 2020; Lee et al., 2020b; Spangler et al., 2015a; Tomala et al., 2009; Trotta et al., 2018; Yokoyama et al., 2018). Here, we built upon a Treg-biasing anti-hIL-2 antibody (Trotta et al., 2018) and engineered a single-chain hIL-2/antibody fusion protein (F5111 IC). Compared with hIL-2/antibody complexes, our IC benefits from extended serum half-life, enhanced stability, and reduced counterproductive activation of Effs. Also, the single-chain format results in a stoichiometrically balanced cytokine-to-antibody ratio and streamlines the clinical development pathway. Indeed, F5111 IC induced a more pronounced Treg bias compared with hIL-2/F5111.2 complex.

Tethering IL-2 to an anti-IL-2 antibody enhances the apparent affinity of the cytokine/antibody interaction (Spangler et al., 2018). However, the mechanism of action for biased immune activation by hIL-2/F5111.2 complex requires cytokine/antibody dissociation. We speculated that the strengthened hIL-2/F5111.2 interaction in the context of the IC would hinder cytokine dissociation, rationalizing our choice to use the weaker affinity F5111 antibody in our IC. In fact, when the F5111.2 antibody was formatted as an IC, Treg activation was severely impaired. Thus, although hIL-2/F5111.2 complex led to enhanced Treg bias compared with the hIL-2/F5111 complex, F5111 outperformed F5111.2 when formatted as ICs.

Based on this finding, as well as the differential IL-2Rα expression patterns between mouse and human immune cells, we designed a panel of F5111 IC variants with varying cytokine/antibody affinities and consequent IL-2 receptor interaction properties. Although cellular studies showed increased Treg activation potency for IC variants, this improved activity was accompanied by increased potency on Effs. The IL-2Rα affinities of IC variants were identical, and discrepancies in IL-2Rβ affinities drove differential immune cell subset engagement. Furthermore, IL-2Rα expression levels were lower within CD8^+^ T and T_Conv_ cell populations activated by IC variants with higher Treg potencies, indicating that these ICs have a lower threshold for activation in the presence of IL-2Rα. Insights from our affinity modulation studies thus provide a roadmap for designing biased IL-2 therapies.

We deployed our IC variants *in vivo*, and a clear cytokine/antibody affinity optimum emerged for the parent F5111 IC, balancing activation of Tregs with concurrent stimulation of Effs. The Treg EC_50_ for F5111 IC was poised at an optimum; enhanced activity on Tregs confounded bias by activating Effs as well, whereas attenuated activity on Tregs led to poor stimulation of all cell types. Importantly, IL-2 receptor expression levels differ between human and mouse cells, and the affinities of receptor subunits toward hIL-2 also vary across species (Spangler et al., 2015a). We observed that the optimal IC variant for biased Treg expansion in humanized mice differed from that in immunocompetent mice, suggesting the possibility for designing patient-specific treatments to address IL-2 receptor level heterogeneity.

F5111 IC design efforts emphasized the importance of optimizing linker length. Shorter linker lengths hindered intramolecular assembly of the cytokine and antibody, driving intermolecular interactions that resulted in higher-order oligomers. Moreover, IC variants with higher Treg potencies eluted as broad single peaks by SEC, whereas F5111.2 IC eluted as a single peak at the expected molecular weight for the monomeric IC, suggesting that stronger cytokine/antibody interactions reduce IC oligomerization. Direct comparison of ICs with intact versus impaired effector function capabilities showed that curbing antibody effector function is critical for avoiding Treg depletion via antibody-dependent cellular cytotoxicity (Saunders, 2019; Wang et al., 2018). Collectively, these findings will guide future IL-2 IC design, and use of the modular hIgG1 scaffold enables extension of the IC approach to other cytokines.

*In vivo* studies showed that F5111 IC improves autoimmune disease outcomes in mice, without compromising pathogen clearance. This represents an important translational achievement for the application of hIL-2-based therapies in autoimmune disease treatment, which is hindered by the cytokine’s short half-life, dosing complications, off-target effects, and toxicity. Deployment of an IL-2-based therapy in a mouse model of immune checkpoint inhibitor-induced diabetes showed that F5111 IC confers long-term protection against drug-induced disease and continues to protect mice even when control mice develop spontaneous disease. F5111 IC had the reverse effect of control IC, which accelerated disease development, likely due to stimulation of Effs. Acceleration of diabetes onset resonates with previous reports that IL-2 and untethered IL-2/antibody complexes can exacerbate disease pathogenesis (Dong et al., 2021; Tang et al., 2008; Wesley et al., 2010), highlighting the safety, selectivity, and efficacy advantages for our IC. Our therapeutic approach could be extended to additional autoimmune conditions, including multiple sclerosis, systemic lupus erythematosus, and GVHD (Klatzmann and Abbas, 2015; Koreth et al., 2011; Webster et al., 2009), and it could also be leveraged to suppress anti-drug immune responses or to prevent immune-related adverse events in cancer patients (June et al., 2017; Kang et al., 2021; Quandt et al., 2021; Stamatouli et al., 2018).

F5111 IC biases the activity of IL-2 while also extending its serum half-life; however, it does not target IL-2 toward specific tissues. Several recent approaches have been taken to target IL-2 and other cytokines to specific locations, such as the tumor microenvironment (Hutmacher et al., 2019; Mortara et al., 2018; Ongaro et al., 2020; Silver et al., 2021). Our approach could be integrated with these emerging technologies to bias the local immune microenvironment and enhance therapy. In addition, F5111 could be incorporated with antigen-specific immune-activating technologies to enable targeted activation of disease-protective Tregs. Overall, this work presents a stable, off-the-shelf Treg-expanding agent with potential applications as a research tool and for therapeutic design.

### Limitations of the study

Certain limitations need to be addressed in future studies of F5111 IC to advance clinical translation. Mechanistic experiments will consider the comparative effects of F5111 IC on various Treg subsets. For instance, detection of Helios and Neuropilin-1 could be used to interrogate the expansion and survival of thymically versus peripherally derived Tregs (Weiss et al., 2012; Yadav et al., 2012). Additional phenotyping and RNA sequencing studies are also needed to compare the functional activity of F5111 IC-expanded Tregs relative to endogenous Tregs. It will also be important to characterize Treg persistence in disease models to better understand the long-term effects of F5111 IC.

We note that autoimmune disease models in this study used prophylactic treatment strategies due to the rapid onset of pathogenesis. Although there is evidence that preventative treatment can be effective in delaying development of type 1 diabetes (Herold et al., 2019) and that prophylactic therapy could offer benefit in rheumatoid arthritis (Dekkers et al., 2017), such early interventions are not always possible. Thus, future work will explore autoimmune disease reversal. We plan to demonstrate generality of F5111 IC through testing in additional contexts, such as GVHD and allograft rejection models and the experimental autoimmune encephalomyelitis model of multiple sclerosis. Furthermore, although the DSS colitis model has been widely used to evaluate IL-2-based therapies (Abo et al., 2019; Lee et al., 2020a; Sagiv et al., 2009; Spangler et al., 2015a), colitis can also be induced in immunodeficient mice lacking T and B cells using this model (Dieleman et al., 1994; Strober et al., 2002). Therefore, it will be important to demonstrate the clinical potential of F5111 IC in other colitis models, such as the chronic T cell transfer model or 2,4,6-trinitrobenzene sulfonic acid-induced colitis.

Finally, to advance clinical development of F5111 IC, it will be important to address developability considerations. For instance, further optimization of sequence and/or linker length may be needed to increase IC purity and yield. Also, it will be critical to benchmark our molecule against other IL-2-based therapies and Treg-biased molecules. Collectively, downstream studies of the activity, therapeutic efficacy, and biophysical properties of F5111 IC will offer valuable insight into its mechanistic activities and support therapeutic translation.

## STAR★METHODS

### RESOURCE AVAILABILITY

#### Lead contact

Further information and requests for resources and reagents should be directed to and will be fulfilled by the lead contact, Jamie Spangler (jamie.spangler@jhu.edu).

#### Materials availability

Reagent generated in this study will be made available on request, but we may require a completed Materials Transfer Agreement.

#### Data and code availability

- All data reported in this paper will be shared by the lead contact upon request.
- This paper does not report original code.
- Any additional information required to reanalyze the data reported in this paper is available from the lead contact upon request.

### EXPERIMENTAL MODEL AND SUBJECT DETAILS

#### Cell lines

HEK 293F cells (ThermoFisher Scientific) were cultivated in Freestyle 293 Expression Medium (ThermoFisher Scientific) supplemented with 2 U/mL penicillin-streptomycin (Gibco). Unmodified YT-1 (Yodoi et al., 1985) and IL-2Rα^+^ (Kuziel et al., 1993) YT-1 human NK cells were cultured in RPMI complete medium (RPMI 1640 medium supplemented with 10% FBS, 2 mM L-glutamine, 1x minimum non-essential amino acids, 1 mM sodium pyruvate, 25 mM HEPES, and 100 U/mL penicillin-streptomycin [Gibco]). All cell lines were maintained at 37°C in a humidified atmosphere with 5% CO_2_.

#### Human PBMCs

For *in vitro* studies containing human PBMCs, leukopaks containing de-identified whole blood were obtained from Anne Arundel Medical Blood Donor Center (Anne Arundel, Maryland, USA). For the immune cell subset expansion studies in humanized mice, PBMCs were isolated from leukocyte cones from de-identified healthy volunteers (NHS Blood and Transport, UK). Human tissue samples taken by NHS Blood and Transport with informed consent and ethical approval from the Oxfordshire Research Ethics Committee, study number 07/H0605/130.

#### Mice

Female NOD/ShiLt and both male and female C57BL/6 mice were purchased from The Jackson Laboratory unless otherwise specified. Mice were used at 8 weeks of age. Animals were housed in specific pathogen-free conditions and experiments conducted in accordance with National Institutes of Health guidelines, and approval by the Johns Hopkins University Animal Care and Use Committee.

For the immune cell subset expansion studies in humanized mice, male BRG mice were obtained from The Jackson Laboratory and were maintained under specific pathogen-free conditions in the Biomedical Services Unit of the University of Oxford (Oxford, United Kingdom). Mice were used at 8 weeks of age. All experiments in this study were performed using protocols approved by the Committee on Animal Care and Ethical Review at the University of Oxford and in accordance with the UK Animals (Scientific Procedures) Act 1986.

For the *in vitro* Treg suppression assay, both male and female C57BL/6 CD45.1 and C57BL/6 FOXP3-IRES-mRFP mice were purchased from The Jackson Laboratory. For the C57BL/6 CD45.1; RFP-FOXP3 mice, C57BL/6 CD45.1 and C57BL/6 FOXP3-IRES-mRFP mice were bred in house to homozygosity and maintained. 8-week-old female C57BL/6 CD45.1; RFP-FOXP3 mice were used for all studies. All mice were housed and bred under specific pathogen-free conditions at Johns Hopkins University Animal Care and Use Facility in Cancer Research Building I. The Institutional Animal Care and Use Facility at Johns Hopkins University approved all animal experiments.

For the comparison of cTreg versus eTreg expansion and the *Toxoplasma gondii* infection mouse model, 6-week-old male C57BL/6 mice were purchased from Taconic Biosciences (Rensselaer, NY, USA) and kept in the University of Pennsylvania Department of Pathobiology vivarium until they reached 8–9 weeks of age for experimental use. CBA/Ca mice were purchased from the Jackson Laboratory and maintained kept in the University of Pennsylvania Department of Pathobiology vivarium. Mice housed in the University of Pennsylvania Department of Pathobiology vivarium were maintained under institutional guidelines of 12-hour light/dark cycles, temperature ranges of 68–77°F and humidity ranging from 35–55%. Ethical oversight of all animal use in this study was approved by the University of Pennsylvania Institutional Animal Care and Use Committee.

For the DSS-induced colitis mouse model, female BALB/c mice were acquired from the colony kept at the Czech Centre for Phenogenomics, Prague, Czech Republic. Mice were used at 8 weeks of age. They were housed and handled according to the institutional committee guidelines with free access to food and water. Animal experiments were approved by the Animal Care and Use Committee of the Institute of Molecular Genetics and were in agreement with local legal requirements and ethical guidelines.

For the immune checkpoint inhibitor-induced diabetes mellitus mouse model, female NOD/ShiLtJ mice were purchased from the Jackson Laboratory and maintained in the UCSF specific pathogen-free animal facility in accordance with guidelines established by the Institutional Animal Care and Use Committee and Laboratory Animal Resource Center. Mice were used at 8 weeks of age.

### METHOD DETAILS

#### Protein purification and expression

The published VH and VL sequences of F5111 (Trotta et al., 2018) were used to formulate the recombinant F5111 antibody on the human IgG1 lambda isotype platform. The heavy chain (HC) and LC of the F5111 antibody were separately cloned into the gWiz vector (Genlantis). Antibodies were expressed recombinantly in HEK 293F cells via transient co-transfection of plasmids encoding the HC and LC. HC and LC plasmids were titrated in small-scale co-transfection tests to determine optimal ratios for large-scale expression. HEK 293F cells were grown to 1.2×10^6^ cells/mL and diluted to 1.0×10^6^ cells/mL. Midiprepped DNA (1 mg total of HC and LC plasmids per liter of cells) and 2 mg per liter of cells of polyethyleneimine (PEI, Polysciences) were independently diluted to 0.05 and 0.1 mg/mL in OptiPro medium (ThermoFisher Scientific), respectively, and incubated at room temperature for 15 minutes. Equal volumes of DNA and PEI were mixed and incubated at room temperature for an additional 15 minutes. Subsequently, the diluted HEK 293F cells and 40 mL/L of DNA/PEI mixture were added to a shaking flask and incubated at 37°C and 5% CO_2_ with rotation at 125 rpm for 5 days. Secreted antibodies were purified from cell supernatants 5 days post-transfection via protein G agarose (ThermoFisher Scientific) affinity chromatography followed by SEC on a Superdex 200 Increase 10/300 GL column (GE Healthcare) on a fast protein liquid chromatography (FPLC) instrument, equilibrated in HEPES-buffered saline (HBS, 150 mM NaCl in 10 mM HEPES pH 7.3). Purity (>99%) was verified by SDS-PAGE analysis.

For F5111 IC production, the hIL-2 cytokine (residues 1–133) was fused at the N-terminus of the F5111 antibody LC, connected by either a flexible 15-amino acid (Gly_4_Ser)_3_ linker (F5111 IC LN15), a 25-amino acid (Gly_4_Ser)_5_ linker (F5111 IC LN25), or a 35-amino acid (Gly_4_Ser)_7_ linker (F5111 IC LN35). We prepared a plasmid encoding the hIL-2-fused F5111 LC into the gWiz vector (Genlantis). ICs were expressed and purified via transient co-transfection of HEK 293F cells with the F5111 HC and the hIL-2-fused F5111 LC plasmids, as described for the F5111 antibody. F5111 IC variants (all with a 35-amino acid linker) were generated in the same manner, with the indicated single-point mutation in either the HC or the hIL-2 fused LC construct. The following single-point mutations were made to the V_H_ sequence: Y35A, Y52A, Y54A, Y60A, and V103A. The following single-point mutations were made to the V_L_ sequence: Y33A, Y94A, and S96A. The sequence for the F5111.2 antibody and F5111.2 IC constructs was obtained from the following patent: (Rondon et al., 2015).

The Control IC was generated in the same manner as described for F5111 IC. Published VH and VL sequences of the FITC-E2 antibody (Honegger et al., 2005) were used to formulate the recombinant Control IC on the human IgG1 lambda isotype platform. hIL-2 (residues 1–133) was fused at the N-terminus of the LC, connected by a 35-amino acid (Gly_4_Ser)_7_ linker. Separate plasmids were constructed in the gWiz vector (Genlantis) encoding the Control HC and the hIL-2-fused Control LC. The Control IC was expressed via transient co-transfection of HEK 293F cells with the HC and hIL-2-fused LC plasmids. Purification proceeded as described for the F5111 antibody.

Antibody or IC constructs with Fc effector function knocked out were generated in the same manner as above, using a HC plasmid with the N297A mutation (Mimura et al., 2000, 2001; Saunders, 2019; Tao et al., 1993; Wang et al., 2018). In all studies, the F5111.2 antibody includes the N297A mutation. All *in vivo* studies were performed with constructs with Fc effector function knocked out unless otherwise noted.

The hIL-2 cytokine (residues 1–133) was cloned into the gWiz vector (Genlantis) with a C-terminal hexahistidine tag. Protein was expressed via transient transfection of HEK 293F cells, as detailed for antibody constructs, and purified via Ni-NTA affinity chromatography followed by SEC using a Superdex 200 Increase 10/300 GL column (GE Healthcare) on an FPLC instrument, equilibrated in HBS. Purity (>99%) was verified by SDS-PAGE analysis.

For expression of biotinylated hIL-2, and the extracellular domains of the hIL-2Rα (residues 1–217) and hIL-2Rβ (residues 1–214) receptor subunits sequences were cloned into the gWiz vector (Genlantis) with a C-terminal biotin acceptor peptide (BAP)-GLNDIFEAQKIEWHE followed by a hexahistidine tag. Proteins were expressed and purified via Ni-NTA affinity chromatography and then biotinylated with the soluble BirA ligase enzyme in 0.5 mM Bicine pH 8.3, 100 mM ATP, 100 mM magnesium acetate, and 500 mM biotin (Avidity). Excess biotin was removed by SEC on a Superdex 200 Increase 10/300 column (GE Healthcare) on an FPLC instrument, equilibrated in HBS. Complete biotinylation was verified via SDS-PAGE streptavidin shift assay.

#### Yeast surface binding studies

For binding studies on yeast, hIL-2 (residues 1–133) or mIL-2 (residues 1–149) were cloned into the pCT3CBN yeast display vector (a variant of pCT302 (Boder and Wittrup, 1997) with an N-terminal yeast agglutinin protein (Aga2) fusion followed by a 3C protease site, a C-terminal myc epitope tag, and BamHI/NotI gene-flanking restriction sites). After induction for 48 hours, 1×10^5^ cells of IL-2-displaying yeast per well were transferred to a 96-well plate and incubated in PBE (PBS with 0.1% BSA and 1 mM EDTA) containing serial dilutions of recombinant F5111 antibody for 2 hours at room temperature. Cells were then washed and stained with anti-human IgG Fc APC (HP6017, BioLegend 409306, 1:50) in PBE for 15 minutes at 4°C. After a final wash, cells were analyzed for antibody binding using a CytoFLEX flow cytometer (Beckman Coulter). Background-subtracted and normalized binding curves were fitted to a first-order binding model, and K_D_ values were determined using GraphPad Prism. Studies were performed three times with similar results.

#### Bio-layer interferometry binding measurements

Binding studies were performed using bio-layer interferometry on an OctetRED96® bio-layer interferometry instrument (Molecular Devices). Biotinylated hIL-2, hIL-2Rα, and hIL-2Rβ were immobilized to streptavidin-coated biosensors (Sartorius) in 0.45 μm filtered PBSA (PBS pH 7.2 containing 0.1% BSA). hIL-2 and hIL-2Rβ were immobilized at a concentration of 50 nM for 120 seconds and hIL-2Rα was immobilized at a concentration of 100 nM for 120 seconds. Once baseline measurements were collected in PBSA, binding kinetics were measured by submerging the biosensors in wells containing serial dilutions of the appropriate analyte for 300 seconds (association) followed by submerging the biosensor in wells containing only PBSA for 600 seconds (dissociation). hIL-2/F5111 or hIL-2/F5111.2 complexes were formed by incubating a 1:1 molar ratio of the F5111 antibody to hIL-2 for 60 minutes at 37°C and then diluting to the appropriate concentration. An irrelevant protein (the monoclonal antibody trastuzumab) was immobilized to a reference streptavidin biosensor for subtraction of non-specific binding. Tips were regenerated in 0.1 M glycine pH 2.7. Data was visualized and processed using the Octet® Data Analysis software version 7.1 (Molecular Devices). Equilibrium titration curve fitting and equilibrium dissociation constant (K_D_) value determination was implemented using GraphPad Prism, assuming all binding interactions to be first order. Experiments were reproduced two times with similar results.

#### YT-1 human NK cell activation studies

Approximately 2×10^5^ IL-2Rα^+^ YT-1 or IL-2Rα^−^ YT-1 cells were plated in each well of a 96-well plate and resuspended in 20 μL of RPMI complete medium containing serial dilutions of either hIL-2, hIL-2/F5111 complexes, or IC. hIL-2/F5111 complexes were formed by incubating a 1:1 molar ratio of the F5111 antibody to hIL-2 for 60 minutes at 37°C and then diluting to the appropriate concentration. Cells were stimulated for 20 minutes at 37°C and immediately fixed by addition of paraformaldehyde (Electron Microscopy Sciences) to a final concentration of 1.5% and incubated for 10 minutes at room temperature. Permeabilization of cells was achieved by resuspension in 200 μL of ice-cold 100% methanol (MilliporeSigma) for 30 minutes at 4°C. Fixed and permeabilized cells were washed twice with PBSA and incubated with anti-pSTAT5 AlexaFluor 647 (pY694, BD Biosciences 562076, 1:50) diluted in 20 μL of PBSA for 2 hours at room temperature. Cells were then washed twice in PBSA and analyzed on a CytoFLEX flow cytometer (BeckmanCoulter). Dose-response curves were fitted to a logistic model and maximum values (E_Max_) and half maximal effective concentration (EC_50_) values were calculated using GraphPad Prism data analysis software after subtraction of the MFI of unstimulated cells and normalization to the maximum signal intensity. Experiments were conducted in triplicate and performed at least twice with similar results.

#### Human PBMC and mouse splenocyte activation studies

For IL-2 induced pSTAT5 assays in human PBMCs, leukopaks containing de-identified whole blood were obtained from Anne Arundel Medical Blood Donor Center (Anne Arundel, Maryland, USA). Human PBMCs were isolated from whole blood by density gradient centrifugation using a standard Ficoll gradient (Ficoll Paque, MilliporeSigma) according to the manufacturer’s protocol. PBMCs were subjected to ACK red blood cell lysis (Quality Biological) and resuspended in PBS. Approximately 2×10^6^ cells/well were then plated into 96-well plate, pelleted, and resuspended in 40 μL of RPMI complete medium containing serial dilutions of the appropriate treatment. hIL-2/antibody complexes were formed by incubating a 1:1 molar ratio of hIL-2 to either the F5111 or F5111.2 antibody for 60 minutes at 37°C and then diluting to the appropriate concentration. Cells were stimulated for 20 minutes at 37°C and immediately fixed by addition of 160 μL of 1× TFP Fix/Perm buffer (Transcription Factor Phospho Buffer Set, BD Biosciences) and incubated at 4°C for 50 minutes. 40 μL 1× TFP Perm/Wash buffer (Transcription Factor Phospho Buffer Set, BD Biosciences) was then added to each well, and cells were then pelleted and washed again with 200 μL of 1× TFP Perm/Wash buffer. Permeabilization was achieved by resuspending the cells in 150 μL of Perm Buffer III (BD Biosciences) and incubating for 30 minutes at 4°C. Cells were then washed with 200 μL of 1× TFP Perm/Wash buffer and then resuspended in 50 μL of 1× TFP Perm/Wash buffer containing the following antibodies: anti-human CD3 APC-eFlour780 (UCHT1, ThermoFisher Scientific 47–0038-42, 1:50), anti-human CD4 PerCp-Cy5.5 (SK3, BD Biosciences 341654, 1:20), anti-human CD8 BV605 (SK1, BioLegend 344742, 1:50), anti-human IL-2Rα BV421 (M-A251, BD Biosciences 562442, 1:100), anti-human FOXP3 PE (236A/E7, BD Biosciences 560852, 1:50), anti-pSTAT5 AlexaFluor 647 (pY694, BD Biosciences 562076, 1:50), and anti-human CD127 Alexa Fluor 488 (eBioRDR5, ThermoFisher Scientific 53–1278-42, 1:50). Cells were incubated for 2 hours at room temperature and then washed twice with PBSA. Data were collected on a BD Biosciences LSRII flow cytometer (Becton Dickinson) and analyzed using FlowJo software (FlowJo, LLC). Tregs were gated as CD3^+^CD4^+^IL-2Rα-^High^FOXP3^High^ cells, CD8^+^ T cells were gated as CD3^+^CD8^+^ cells, and T_Conv_ cells were gated as CD3^+^CD4^+^ FOXP3^−^ cells. pSTAT5 dose-response curves were fitted to a logistic model and E_Max_ and EC_50_ values were calculated using GraphPad Prism data analysis software after subtraction of the MFI of unstimulated cells and normalization to the maximum signal intensity. Unless otherwise specified, PBMC activation experiments were conducted in triplicate and performed at least twice using PBMCs from independent donors.

For IL-2-induced pSTAT5 assays in murine lymphocytes, spleens from female NOD/ShiLtJ mice (The Jackson Laboratory) were collected and processed into a single-cell suspension followed by ACK red blood cell lysis (Quality Biological). Cells were seeded at 2×10^6^ cells/well into a 96-well plate, and the same protocol as for PBMC studies was followed using a different panel of antibodies: anti-mouse CD3 BV510 (145–2C11, BioLegend 100353, 1:100), anti-mouse CD4 APC-eF780 (RM4–5, ThermoFisher Scientific 47-0042-82, 1:100), anti-mouse CD8a AlexaFluor 488 (53–6.7, BD Biosciences 557668, 1:50), anti-mouse IL-2Rα PE (PC61.5, ThermoFisher Scientific 12-0251-83, 1:100), anti-mouse FOXP3 eFlour450 (FJK-16s, ThermoFisher Scientific 48-5773-82, 1:50), and anti-pSTAT5 Alexa Fluor 647 (pY694, BD Biosciences 562076, 1:50). Mouse splenocyte activation studies were conducted in triplicate.

#### Quantification of IL-2Rα expression levels

Human PBMCs were isolated as described in the section “human PBMC and mouse splenocyte activation studies.” Approximately 2×10^6^ human PBMCs/well or 0.2×10^6^ YT-1 cells/well were resuspended in 50 μL of PBS containing LIVE/DEAD^™^ Fixable Blue Dead Cell Stain Kit (ThermoFisher Scientific L34961, 1:1000) and stained for 15 minutes at 4°C. Cells were washed with PBSA, resuspended in 40 μL of RPMI, and then fixed by addition of 160 μL of 1× TFP Fix/Perm buffer (Transcription Factor Phospho Buffer Set, BD Biosciences) and incubated at 4°C for 50 minutes. 40 μL 1× TFP Perm/Wash buffer (Transcription Factor Phospho Buffer Set, BD Biosciences) was then added to each well, and cells were then pelleted and washed again with 200 μL of 1× TFP Perm/Wash buffer. Permeabilization was achieved by resuspending the cells in 150 μL of Perm Buffer III (BD Biosciences) and incubating for 30 minutes at 4°C. Cells were then washed with 200 μL of 1× TFP Perm/Wash buffer and resuspended in 50 μL of 1× TFP Perm/Wash buffer containing the following antibodies: anti-human CD3 APC-eFlour780 (UCHT1, ThermoFisher Scientific 47-0038-42, 1:50), anti-human CD4 PerCp-Cy5.5 (SK3, BD Biosciences 566316, 1:100), anti-human CD8 BV605 (SK1, BioLegend 344742, 1:50), anti-human IL-2Rα BV421 (M-A251, BD Biosciences 562442, 1:100), and anti-human FOXP3 PE (236A/E7, BD Biosciences 560852, 1:50). For the IL-2Rα FMO control, the same panel was used minus the addition of anti-human IL-2Rα BV421. Cells were incubated for 2 hours at room temperature and then washed twice with PBSA. Data were collected on a BD Biosciences LSRII flow cytometer (Becton Dickinson) and analyzed using FlowJo software (FlowJo, LLC). Staining was conducted in triplicate and performed twice using PBMCs from independent donors.

#### Development of a multivalent binding model for IC signaling

The multivalent binding model used to predict cell type-specific signaling response to ICs was formulated as described in Tan et al. (Tan and Meyer, 2021). Each IL-2 molecule within the IC was assumed to bind to one free IL-2Rα and one IL-2Rβ/γ_c_ receptor; therefore, bivalent ICs were allowed to bind up to two IL-2Rα and IL-2Rβ/γ_c_ receptors each. Initial IL-2-IL-2Rα association was modeled as proceeding with the experimentally determined kinetics of monomeric ligand-receptor interaction. The affinities with which each IC initially interacted with IL-2Rβ/γ_c_ receptor dimer were inferred by fitting the binding model to our experimental *in vitro* pSTAT5 signaling data as described below, using least-squares fitting. Subsequent ligand-receptor binding interactions were modeled with an association constant proportional to the free receptor abundance and the monomeric affinity of receptor-ligand interaction multiplied by the scaling constant,$K_{x}^{*}$. A single $K_{x}^{*}$ value was fit for all experiments and cell types when we fit our model to our *in vitro* pSTAT5 signaling data. To predict pSTAT5 response to IL-2 stimulation, we assumed that pSTAT5 is proportional to the amount of IL-2-bound IL-2Rβ/γ_c_, as complexes which contain these species actively signal through the JAK/STAT pathway. Scaling factors converting from predicted active signaling species to pSTAT5 abundance were fit to experimental data on a per-experiment and cell type basis. The abundance of each IL-2 receptor subunit on each cell type was assumed to be equal to previously published experimental human PBMC receptor quantitation data (Farhat et al., 2021).

#### Immune cell subset expansion studies in NOD mice

For immune cell expansion studies in NOD mice (Tomala and Spangler, 2020), 8-week-old female NOD/ShiLtJ mice (4–5 mice per group, The Jackson Laboratory) were injected intraperitoneally (*i.p.*) for 4 consecutive days (Days 0, 1, 2, 3) with either 200 μL of PBS or the indicated treatment diluted in 200 μL of PBS. ICs were dosed at 8.2 μg per day (1.5 μg hIL-2 equivalence) and hIL-2/F5111.2 antibody complexes were formed by preincubating 1.5 μg hIL-2 with 6.6 μg F5111.2 antibody (1:2 antibody to cytokine molar ratio) in PBS for 60 min at 37°C. Mice were sacrificed 24 hours after the last dose (Day 4) by cervical dislocation, and spleens were harvested. Single-cell suspensions were prepared by mechanical homogenization and then subjected ACK red blood cell lysis (Quality Biological) and resuspended in PBS. Absolute count of splenocytes was assessed for each spleen. Approximately 2×10^6^ cells were used per sample. To assess viability, cells were resuspended in 50 μL of PBS containing eBioscience Fixable Viability Dye eFluor780 (ThermoFisher Scientific 65-0865-18, 1:2000) and stained for 15 minutes at 4°C. Cells were then washed with PBSA and resuspended in 50 μL of PBSA containing the following antibodies: anti-mouse CD3 BV510 (145–2C11, BioLegend 100353, 1:50), anti-mouse CD4 eFluor450 (RM4–5, ThermoFisher Scientific 48-0042-82, 1:100), anti-mouse CD8a AlexaFluor 488 (53–6.7, BD Biosciences 557668, 1:50), anti-mouse IL-2Rα PE (PC61.5, ThermoFisher Scientific 12-0251-83, 1:100), anti-mouse CD49b PE-Cy7 (DX5, BioLegend 108992, 1:100), and anti-mouse CD16/CD32 (2.4G2, BD Biosciences 553142, 1:100). Cells were stained for 30 minutes at 4°C. Cells were then washed with PBSA and resuspended in 200 μL of 1× eBioscience Fixation/Permeabilization buffer (ThermoFisher Scientific) and incubated for 45 minutes at 4°C. 800 μL of 1× eBioscience Permeabilization buffer (ThermoFisher Scientific) was then added to each tube. Cells were subsequently resuspended in 50 μL of 1× eBioscience Permeabilization buffer containing anti-mouse FOXP3 APC (FJK-16s, ThermoFisher Scientific 17-5733-82, 1:80) and incubated for 45 minutes at 4°C. After a final wash and resuspension in PBSA, data were collected on a BD Biosciences LSRII flow cytometer (Becton Dickinson) and analyzed using FlowJo software (FlowJo, LLC). Tregs were gated as CD3^+^CD4^+^IL-2Rα^High^FOXP3^High^ cells, CD8^+^ T cells were gated as CD3^+^CD8^+^ cells, T_Conv_ cells were gated as CD3^+^CD4^+^FOXP3^−^ cells, and NK cells were gated as CD3^−^CD49b^+^ cells. Statistical significance was determined by one-way ANOVA with Tukey post hoc test. Experiments were performed at least twice with similar results.

To quantify cell proliferation the same procedure as above was followed using the following panel: eBioscience Fixable Viability Dye eFluor780 (ThermoFisher Scientific 65-0865-18, 1:2000), CD3 BV510 (145–2C11, BioLegend 100353, 1:50), anti-mouse CD4 eFluor450 (RM4–5, ThermoFisher Scientific 48-0042-82, 1:100), anti-mouse CD8a BV570 (53–6.7, BioLegend 100739, 1:100), anti-mouse IL-2Rα PE (PC61.5, ThermoFisher Scientific 12-0251-83, 1:100), anti-mouse CD49b PE-Cy7 (DX5, BioLegend 108992, 1:100), anti-mouse CD16/CD32 (2.4G2, BD Biosciences 553142, 1:100), anti-mouse FOXP3 APC (FJK-16s, ThermoFisher Scientific 17-5733-82, 1:80), anti-mouse Ki-67 AlexaFluor 488 (16A8, BioLegend 652417, 1:100), anti-mouse CD44 PerCP-Cy5.5 (IM7, ThermoFisher Scientific 45-0441-82, 1:100), and anti-mouse Helios PE/Dazzle 594 (22F6, BioLegend 137231, 1:100). Statistical significance was determined by one-way ANOVA with Tukey post hoc test. Experiments were performed at least twice with similar results.

#### Non-negative matrix factorization of immune cell subset expansion studies in NOD mice

The *in vivo* proliferative responses induced by the ICs were visualized using non-negative matrix factorization as implemented in scikit-learn (Pedregosa et al., 2011). The replicate average number of cells measured in NOD mice after treatment with each complex or IC was assembled. The log of the experimentally determined cell number was then taken, and number of each cell type measured during control (PBS) trials was subtracted from all other experimental measurements to obtain the log-fold changes in cell counts induced by each ligand in relation to the control trial. Non-negative matrix factorization with two components was subsequently performed on the processed matrix of cellular expansion data.

#### Immune cell subset expansion studies in humanized mice

Human PBMCs (NHS Blood and Transport) were dyed with Violet Proliferation Dye 450 (BD Biosciences) before *i.p.* injection into 8-week-old male BRG mice (The Jackson Laboratory). Mice received 5×10^6^ PBMCs on day 0 and on day 1 received a single *i.p*. dose of either PBS (n = 6) or 8.2 μg (1.5 μg IL-2 equivalence) Y60A IC (n = 5), Y33A IC (n = 4), F5111 IC (n = 4), or F5111.2 IC (n = 5). Mice were sacrificed on day 4 and the PBMCs were retrieved by peritoneal lavage for flow cytometry analysis.

Cell were stained with Zombie NIR Fixable Dye (BioLegend 423105, 1:8000), anti-mouse CD45 APC-Cy7 (QA17A26, BioLegend 157617, 1:400), anti-mouse TER-119 APC-Cy7 (TER-119, BioLegend 116223, 1:400), anti-human CD3 BV605 (OKT3, BioLegend 317321, 1:200), anti-human CD56 PE (TULY56, ThermoFisher Scientific 12-0566-41, 1:200), anti-human CD4 PE-eFluor610 (RPA-T4, ThermoFisher Scientific 61-0049-42, 1:200), anti-human CD8 BV711 (SK1, BioLegend 344733, 1:200), anti-human IL-2Rα PE-Cy7 (M-A251, BD Biosciences 557741, 1:200), anti-human FOXP3 (259D, BioLegend 320214, 1:200), and anti-human Ki-67 FITC (20Rag1, ThermoFisher Scientific, 1:200). The eBioscience Foxp3/Transcription Factor Staining Buffer Set (ThermoFisher Scientific) was used for intracellular staining following the manufacturers protocol. Samples were collected on an Attune NxT flow cytometer and analyzed using FlowJo software (FlowJo, LLC). Tregs were gated as CD3^+^CD4^+^IL-2Rα^High^FOXP3^High^ cells, CD8^+^ T cells were gated as CD3^+^CD8^+^ cells, and T_Conv_ cells were gated as CD3^+^CD4^+^FOXP3^−^ cells. Murine cell populations (mCD45^+^mTER-119^+^) were excluded from the gating. Statistical significance was determined by one-way ANOVA with Tukey post hoc test. The experiment was performed twice with similar results.

#### Immune cell subset expansion study dose titrations

8-week-old female C57BL/6 mice (2 mice per group, The Jackson Laboratory) were administered either 200 μL of PBS or increasing doses of F5111 diluted in 200 μL of PBS via *i.p*. injection daily for 4 days. The doses of F5111 IC used were 0.91 μg (0.167 μg IL-2 equivalence), 2.7 μg (0.5 μg IL-2 equivalence), 4.1 μg (0.75 μg IL-2 equivalence), 6.2 μg (1.125 μg IL-2 equivalence), and 8.2 μg (1.5 μg IL-2 equivalence). Mice were euthanized 24 hours after the last dose and spleens were harvested. Splenocytes were isolated by mechanical dissociation through 100 μm filters and subjected to ACK red blood cell lysis buffer. Cells were stained with anti-mouse CD45 PerCP-Cy5.5 (30-F11, BioLegend 103132, 1:1200), anti-mouse CD3 BV605 (17A2, BioLegend 100237, 1:80), anti-mouse CD4 APC (GK1.5, BioLegend 100411, 1:3200), anti-mouse CD8a BV786 (53–6.7, BD Biosciences 563332, 1:400), anti-mouse IL-2Rα BV421 (7D4, BD 564571, 1:200). Transcription Factor Phospho Buffer Set (BD) was used for intracellular staining following the manufacturers protocol and then stained with anti-mouse FOXP3 FITC (FJK-16s, ThermoFisher Scientific 11-5773-82, 1:200). Samples were acquired on a BD Celesta and analyzed using FlowJo (FlowJo, LLC). Tregs were gated as CD45^+^CD3^+^CD4^+^IL-2Rα-^High^FOXP3^High^ cells. Statistical significance was determined by one-way ANOVA with Tukey post hoc test.

#### Immune cell subset expansion study kinetics

8-week-old male C57BL/6 mice (3 mice per group, The Jackson Laboratory) were administered *i.p.* injections for 4 consecutive days (Days −3, −2, −1, 0) containing either 200 μL of PBS or 8.2 μg F5111 IC (1.5 μg hIL-2 equivalence) diluted in 200 μL of PBS. Spleens from the PBS-treated group were harvested one day post-treatment (Day 1). Spleens from the F5111 IC treated mice were harvested 1, 3, 5, and 7 days post-treatment (3 mice per harvest). The same protocol was implemented as described for the “Immune cell subset expansion studies in NOD mice” with the substitution of the following antibody panel: anti-mouse CD3 BV510 (145–2C11, BioLegend 100353, 1:100), anti-mouse NK-1.1 PerCp-Cy5.5 (PK136, BioLegend 108727, 1:100), anti-mouse CD4 eFluor450 (RM4–5, ThermoFisher Scientific 48-0042-82, 1:100), anti-mouse CD8a AlexaFluor 488 (53–6.7, BD Biosciences 557668, 1:100), anti-mouse IL-2Rα PE (PC61.5, ThermoFisher Scientific 12-0251-83, 1:100), anti-mouse FOXP3 APC (FJK-16s, ThermoFisher Scientific 17-5733-82, 1:80), and anti-mouse CD16/CD32 (2.4G2, BD Biosciences 553142, 1:100). Tregs were gated as CD3^+^CD4^+^IL-2Rα^High^FOXP3^High^ cells, CD8^+^ T cells were gated as CD3^+^CD8^+^ cells, T_Conv_ cells were gated as CD3^+^CD4^+^FOXP3^−^ cells, and NK cells were gated as CD3^−^NK-1.1^+^ cells.

#### Pharmacokinetic study

F5111 IC was labeled using 10-fold molar excess of N-hydroxysuccinimide (NHS)-Rhodamine (ThermoFisher Scientific), following the manufacturer’s protocol. Non-reacted NHS-rhodamine was removed by SEC on a Superdex 200 Increase 10/300 GL column (GE Healthcare), equilibrated in HBS. Protein concentration and degree of labeling were calculated according to the manufacturer’s protocol.

Prior to treatment, a small volume of blood was collected from the tail vein of 8-week-old male C57BL/6 mice (5 mice, The Jackson Laboratory). Mice were then administered retro-orbital injections of 2 mg/kg (~0.36 mg/kg hIL-2 equivalence, ~10 μg hIL-2 total/mouse) F5111 IC diluted in 200 μL of PBS. Blood was collected from the tail vein of each mouse at 5 minutes, 20 minutes, 40 minutes, 1 hour, 2 hours, 4 hours, 8 hours, 24 hours, 48 hours, 72 hours, 96 hours, and 120 hours. At each time point, blood was collected in EDTA-coated tubes and centrifuged at 500×g for 5 minutes. The plasma was collected and stored at 4°C for later analysis. After all samples were collected, the plasma was diluted in PBS (1:10 dilution), and 100 μL of diluted sample was added to a 96 well black clear-bottom plate. Fluorescence (Excitation/Emission: 540/590 nm) was measured on a BioTek Synergy 2 instrument, and blood samples collected before treatment were used for background subtraction. Standard curves were generated from fluorescent measurements of rhodamine-labeled F5111 IC at concentrations ranging from 3 μg/mL to 0.023 μg/mL (2-fold dilutions) and this standard curve was used to determine protein concentration of the collected samples. Serum half-life was calculated using a two-phase decay model in GraphPad Prism.

#### *In vitro* Treg suppression assay

8-week-old female C57BL/6 CD45.1; RFP-FOXP3 mice (The Jackson Laboratory) were treated with either 200 μL of PBS (n = 3) or 6.2 μg F5111 IC in 200 μL of PBS (n = 2) *i.p*. every day for 4 days. 24 hours after the last dose, Tregs were isolated from spleens from each group for the Treg suppression assay. Spleens from female C57BL/6 CD45.2 mice (n = 5) were taken for isolation of T_Conv_ cells.

Pooled mouse spleens were digested and CD4 cells were positively selected from all mouse groups (Miltenyi, 130-117-043). CD4^+^ cell suspensions were stained with either a Treg isolation panel: anti-mouse CD45.1 APC-Cy7 (A20, BioLegend 110716, 1:400), anti-mouse CD45.2 PerCp-Cy5.5 (104, BioLegend 109828, 1:400), anti-mouse CD4 APC (GK1.5, BioLegend 100411, 1:3200), anti-mouse CD8a BV786 (53–6.7, BD 563332, 1:400), anti-mouse IL-2Rα BV421 (7D4, BD 564571, 1:200), or a T_Conv_ isolation panel: anti-mouse CD45.1 APC-Cy7 (A20, BioLegend 110716, 1:400), anti-mouse CD45.2 PerCp-Cy5.5 (104, BioLegend 109828, 1:400), anti-mouse CD4 APC (GK1.5, BioLegend 100411, 1:3200), anti-mouse CD8a BV786 (53–6.7, BD 563332, 1:400), anti-mouse IL-2Rα BV421 (7D4, BD 564571, 1:200), anti-mouse CD62L PE (MEL-14, BioLegend 104408, 1:200), and anti-mouse CD44 AlexaFluor 700 (IM7, BioLegend 103026, 1:400). CD45.1^+^CD4^+^CD8^−^IL-2Rα^High^RFP-FOXP3^+^ Tregs were sorted from F5111 treated and PBS treated mice. CD45.2^+^CD4^+^CD8^−^IL-2Rα^−^CD62L^High^CD44^Low^ naïve T_Conv_ cells were sorted for effector cells. Cell sorting occurred on a BD FACSAria^™^.

For division tracking, T_Conv_ cells were labeled with CellTrace^™^ Violet (CTV, ThermoFisher Scientific) by incubating cells for 16 minutes at 37°C at a cell density of 10^6^/mL in PBSA. Cells were vortexed every 4 minutes of staining. Reaction was quenched in ice cold RPMI 1640 medium (Invitrogen) with 10% FBS (MilliporeSigma).

Cells were cultured in RPMI 1640 medium (Invitrogen) supplemented with non-essential amino acids (Gibco), 1 mM sodium pyruvate (MilliporeSigma), 10 mM HEPES (Gibco), 100 U/mL Penicillin, 100 μg/mL streptomycin (Quality Biologic), 50 μM 2-mercaptoethanol (Gibco), 2 mM L-glutamine (Gibco) and 10% FBS (MilliporeSigma). Cells were incubated at 37°C in 5% CO_2_. The Treg suppression assay was performed as follows: 50,000 T_Conv_ cells were co-cultured with a varying ratio of Tregs in 96-well U-bottom plate. Anti-CD3/anti-CD28 coated microbeads (Dynabeads Mouse T Activator, ThermoFisher Scientific, 11452D) were added at a 1:2 (bead: cell) ratio with final number of cells in each well. Proliferation was analyzed by flow cytometry done on a BD FACSCelesta^™^ after 4 days. Cells were washed in PBS and stained with anti-mouse CD45.1 APC-Cy7 (A20, BioLegend 110716, 1:400) and anti-mouse CD45.2 PerCp-Cy5.5 (104, BioLegend 109828, 1:400) to differentiate T_Conv_ cells from Tregs. A minimum number of 20,000 CD45.2^+^CTV^+^ cells were collected and analyzed with FlowJo software (FlowJo, LLC). The percent suppression is calculated using the following formula: ((%CTV^−^ of T_Conv_ cells alone - %CTV^−^ of T_Conv_ cells treated with Treg)/%CTV^−^ of T_Conv_ cells alone)*100 (Collison and Vignali, 2011). CTV is gated on unstimulated T_Conv_ with all divisions beyond unstimulated condition considered CTV^−^. Experiment was conducted with technical replicates and repeated twice with similar results. Statistical significance at each ratio was determined by two-tailed unpaired Student *t* test.

#### Comparison of cTreg versus eTreg expansion

8-week-old male C57BL/6 mice (5 mice per group, Taconic Biosciences) were injected *i.p.* every 48 hours (days 0, 2, and 4) with either 200 μL of 1× Dulbecco’s PBS (DPBS, Corning) or 8.2 μg (1.5 μg IL-2 equivalence) Control IC or F5111 IC diluted in 200 μL of 1× DPBS. The mice were sacrificed on day 6 and their splenocytes were harvested for analysis via flow cytometry. Splenocyte single cell suspensions were isolated by mechanically processing harvested spleens through 70 μm nylon filters. The suspensions were spun down at 300×g for 5 minutes, and then resuspended in 1 mL of lysis buffer (0.846% solution of NH_4_Cl) for 5 minutes to lyse red blood cells. The cells were then washed in 10 mL of complete RPMI and then resuspended to 30e^6^ cells/mL on ice for staining.

5e^6^ cells per sample were placed in a 96 well round bottom plate and washed with ice cold 1× DPBS. The cells were resuspended in 50 μL of Ghost Dye^™^ Violet 510 viability dye (Tonbo Biosciences 13-0870-T100, 1:200) reconstituted in 1× DPBS for 20 minutes on ice and then washed in 0.2% FACS buffer (1× DPBS, 2 g/L BSA, 0.5 M EDTA). The cells were then resuspended in 50 μL of volume of 0.2% FACS buffer containing anti-mouse CD16/CD32 (2.4G2, Bio X Cell BP0307, 1 μg/mL) and rat IgG Isotype Control (ThermoFisher Scientific 10700, 1:200) for 30 minutes on ice. The cells were washed in 0.2% FACS buffer, and then incubated for 30 minutes on ice in 50 μL volume of antibody cocktail composed of: anti-mouse CD4 BUV563 (GK1.5, BD Biosciences 612923, 1:200), anti-mouse CD8a BUV615 (53–6.7, BD Biosciences 613004, 1:200), anti-mouse CD11a BUV805 (2D7, BD Biosciences 741919, 1:200), anti-mouse IL-2Rα BV785 (PC61, BioLegend 102051, 1:200), anti-mouse CD27 BV650 (LG.3A10, BioLegend 124233, 1:200), anti-mouse CD44 BV570 (1M7, BioLegend 103037, 1:100), anti-mouse CD69 BUV737 (H1.2F3, BD Biosciences 612793, 1:200), anti-mouse IL-2Rβ BUV661 (TM-β1, BD Biosciences 741493, 1:200), anti-mouse KLRG1 BUV395 (2F1, BD Biosciences 740279, 1:300), anti-mouse ICOS APC/Fire 750 (C398.4A, BioLegend 313536, 1:200), and anti-mouse PD-1 BV421 (29F.1A12, BioLegend 135218, 1:250) in 0.2% FACS buffer supplemented with Brilliant Stain Buffer (BD Biosciences, 1:10). The cells were washed in 0.2% FACS buffer and re-suspended in 100 μL 1× eBioscience Fixation/Permeabilization buffer (ThermoFisher Scientific) for 4 hours at 4°C. The cells were then washed twice in 1× eBioscience Permeabilization buffer (ThermoFisher Scientific), and then resuspended in 50 μL of 1× eBioscience Permeabilization buffer containing: anti-mouse BCL-2 AlexaFluor 647 (BCL/10C4, BioLegend 633510, 1:200), anti-mouse CD3 BV750 (17A2, BioLegend 100249, 1:200), anti-mouse CTLA-4 APC-R700 (UC10-4F10-11, BD Biosciences 565778, 1:300), anti-mouse FOXP3 PE-Cy5.5 (FJK-16s, ThermoFisher Scientific 35-5773-82, 1:200), anti-mouse Helios PE/Dazzle 594 (22F6, BioLegend 137231, 1:200), anti-mouse Ki-67 AlexaFluor 488 (B56, BD Biosciences 558616, 1:400), and anti-mouse T-bet PE-Cy5 (4B10, ThermoFisher Scientific 15-5825-82, 1:200) for 2 hours at 4°C. The cells were then washed with 1× eBioscience Permeabilization buffer twice, and then resuspended in 500 μL 0.2% FACS buffer for flow cytometric analysis. Fluorescence minus one (FMOs) controls and individual single color controls and were created using pooled remaining splenocytes and were prepared simultaneously with experimental samples. The prepared and stained cells were analyzed using a FACS Symphony A5 (BD Biosciences) running BD FACSDiva v9.0 (BD Biosciences). Raw flow cytometry data was compensated and analyzed using FlowJo (FlowJo, LLC). cTregs were gated as CD4^+^FOXP3^+^IL-2Rα^High^BCL-2^High^ and eTregs were gated as CD4^+^FOXP3^+^IL-2Rα^Low^BCL-2^Low^. Statistical significance was determined separately for cTregs and eTregs by one-way ANOVA with a Tukey post hoc test.

#### *Toxoplasma gondii* infection mouse model

ME49 strain *Toxoplasma gondii* cysts were obtained from neural tissue harvested from chronically infected CBA/Ca mice (The Jackson Laboratory) and resuspended to 125 cysts/mL in 1× DPBS (Corning). Cohorts of male C57BL/6 mice (5 mice per group, Taconic Biosciences) were infected via *i.p*. injection at 8–9 weeks of age using 200 μL of diluted ME49 cysts for an infection dose of 25 cysts/mouse. The disease-free control group did not receive ME49 cysts and instead received an injection of 1× DPBS. Mice were then injected *i.p*. with either 200 μL of 1× DPBS (control group and PBS) or 8.2 μg (1.5 μg IL-2 equivalence) Control IC or F5111 IC diluted in 200 μL of 1× DPBS every 24 hours after infection on days 1, 2, 3, 4, and 5. The mice were sacrificed on day 10 of infection, and splenocytes were harvested for analysis via flow cytometry, the right lateral lobe of the lungs and liver were frozen at −80°C for DNA isolation, and the left lateral lobe of the liver was stored in 10% buffered formalin (Jansen Pharmaceuticals) for histological analysis.

The staining and flow cytometry analysis was performed as described in the “Comparison of cTreg versus eTreg expansion” methods except anti-mouse CD27 was excluded and the following antibodies were added to the panel: anti-mouse CXCR3 BV650 (CXCR3–173, BioLegend 126531, 1:150), anti-mouse NK-1.1 BV711 (PK136, BioLegend 108245, 1:300), and anti-mouse NKp46 BV605 (29A1.4, BioLegend 137619, 1:300). Additionally, toxoplasma-specific tetramers for CD4^+^ (Tetramer I-A(b) AS15 PE, NIH, AVEIHRPVPGTAPPS, 1:400) and CD8^+^ (Tetramer H2k(b) Tgd057 PE, NIH, SVLAFRRL, 1:400) T cells were included. Tregs were gated as CD3^+^CD4^+^IL-2Rα^High^FOXP3^High^ cells, CD8^+^ T cells were gated as CD3^+^CD8^+^ cells, and T_Conv_ cells were gated as CD3^+^CD4^+^FOXP3^−^ cells.

Harvested left lateral liver lobes were kept submerged in 10% buffered formalin for 48 hours then mounted in paraffin, cut, and hematoxylin and eosin (H&E) stained by the University of Pennsylvania Comparative Pathology Core.

Total DNA was isolated from frozen sections of the liver, heart, and lungs using a DNeasy Blood and Tissue Kit (Qiagen) according to manufacturer protocol. Isolated DNA concentration was quantified via NanoDrop spectrophotometry (ThermoFisher Scientific) and then individual samples were diluted in DNase/RNase-free distilled water (ThermoFisher Scientific) to equivalent concentrations for qPCR analysis (lungs 40 ng/μL, livers 50 ng/μL). Parasite DNA quantity was assessed in triplicate from the individual prepared DNA samples via qPCR using toxoplasma specific primers: (forward) 5′-TCCCCTCTGCTGGCGAAAAGT-3′ and (reverse) 5′-AGCGTTCGTGGTCAACTATCGATTG-3′ with Power SYBR Green master mix (ThermoFisher Scientific). The reaction conditions for the qPCR were: holding phase of 2 minutes at 50°C, and then 10 minutes at 95°C (occurs only once); followed by 50 cycles of PCR phases of 15 seconds at 95°C, and 1 minute at 60°C. The qPCR reaction was performed on a ViiA 7 Real-Time PCR system operating ViiA 7™ Software. Analysis of parasite qPCR data was performed in Microsoft Excel version 2202 (Build 14931.20120).

Statistical significance was determined by one-way ANOVA with Tukey post hoc test. The experiment was performed twice with similar results.

#### DSS–induced colitis mouse model

Female, 8-week-old, BALB/c mice (6 mice per group, Czech Centre for Phenogenomics) were injected *i.p.* daily for 7 days (days 0–6) with either 200 μL of PBS (control group and PBS) or 8.2 μg (1.5 μg hIL-2 equivalence) Control IC or F5111 IC diluted in 200 μL of PBS. For the complex, 1.5 μg hIL-2 was complexed with 6.6 μg F5111.2 antibody (1:2 antibody to cytokine molar ratio) in 200 μL of PBS for 60 min at 37°C. Beginning on day 7, all groups except for the disease-free control group were administered 3% DSS (MW = 36,000–50,000; MP Biomedicals) in their drinking water to induce colitis. Mice weight, stool consistency, and rectal bleeding were measured daily and scores were assigned for each category (Cooper et al., 1993). The weight loss score was calculated based on initial weight as follows: 0 (<1% weight loss); 1 (1–5% weight loss); 2 (5–10% weight loss); 3 (10–20% weight loss); 4 (>20% weight loss). Stool consistency was scored as follows: 0 (normal stool); 2 (loose stool); 4 (diarrhea/liquid stool). Rectal bleeding was scored as follows: 0 (no presence of blood); 4 (blood observed). The disease activity index was calculated as the average of the weight loss, stool consistency, and rectal bleeding. On day 15, mice were sacrificed and entire colons were removed (from cecum to anus). Colon length was measured, and shortening was used as an indirect marker of pathological inflammation. Distal colon sections were fixed in Carnoy’s solution and embedded in paraffin. Histological scoring of paraffin-embedded and hematoxylin and eosin-stained transversal colon sections was implemented in a blinded manner using a weighted score, ranging from 0 (no signs of inflammation) to 4 (severe inflammation). Statistical significance was determined by one-way ANOVA with Tukey post hoc test. The experiment was performed two times with similar results.

#### Immune checkpoint inhibitor-induced diabetes mellitus mouse model

8-week-old female NOD/ShiLtJ mice (the Jackson Laboratory) were treated *i.p*. with either 200 μL of PBS (control group and PBS) or 8.2 μg (1.5 μg IL-2 equivalence) Control IC or F5111 IC diluted in 200 μL of PBS from day −3 to 0 prior to initiating anti-PD-1 treatment. Starting on day 0 (4 hours after the last IC treatment), mice were treated *i.p*. every 4 days with either PBS (control group) or 200 μg anti-PD-1 antibody (clone RMP1–14, Bio X Cell) for a total of four or five doses. Non-fasting blood glucose was monitored by using a OneTouch® Ultra® 2 glucometer. Diabetes onset was considered to have occurred when non-fasting blood glucose concentration exceeded 250 mg/dL for two consecutive measurements. Statistical significance was determined by pairwise comparisons using the Log-rank (Mantel-Cox) test.

### QUANTIFICATION AND STATISTICAL ANALYSIS

All statistical analysis was performed using GraphPad Prism. The number of replicates, number of mice, definition of center, dispersion and precision measures, and type of analysis performed is described in each of the above sections where applicable. p ≤ 0.05 was considered significant for all experiments (*p ≤ 0.05, **p ≤ 0.01, ***p ≤ 0.001, ****p ≤ 0.0001). Significance between all groups is not always shown in figures, for full analysis see Table S6.

## Supplementary Material

1

2

## Figures and Tables

**KEY RESOURCES TABLE:** 

REAGENT or RESOURCE	SOURCE	IDENTIFIER

Antibodies		

F5111 Antibody	This manuscript; see Table S1	N/A
F5111.2 Antibody (N297A)	This manuscript; see Table S1	N/A
Trastuzumab Antibody	This manuscript	N/A
Anti-human IgG Fc APC [HP6017]	BioLegend	Cat# 409306; RRID: AB_11149491
Anti-mouse/human phospho-STAT5 AlexaFluor 647 [pY694]	BD Biosciences	Cat# 562076; RRID: AB_11154412
Anti-human CD3 APC-eFluor780 [UCHT1]	ThermoFisher Scientific	Cat# 47-0038-42; RRID: AB_1272042
Anti-human CD4 PerCP-Cy5.5 [SK3]	BD Biosciences	Cat# 341654; RRID: AB_400452
Anti-human CD8 BV605 [SK1]	BioLegend	Cat# 344742; RRID: AB_2566513
Anti-human IL-2Rα BV421 [M-A251]	BD Biosciences	Cat# 562442; RRID: AB_11154578
Anti-human FOXP3 PE [236A/E7]	BD Biosciences	Cat# 560852; RRID: AB_10563418
Anti-human CD127 AlexaFluor 488 [eBioRDR5]	ThermoFisher Scientific	Cat# 53-1278-42; RRID: AB_2744750
Anti-mouse CD3 BV510 [145-2C11]	BioLegend	Cat# 100353; RRID: AB_2565879
Anti-mouse CD4 APC-eF780 [RM4-5]	ThermoFisher Scientific	Cat# 47-0042-82; RRID: AB_1272183
Anti-mouse CD8a AlexaFluor 488 [53-6.7]	BD Biosciences	Cat# 557668; RRID: AB_396780
Anti-mouse IL-2Rα PE [PC61.5]	ThermoFisher Scientific	Cat# 12-0251-83; RRID: AB_465608
Anti-mouse FOXP3 eFluor450 [FJK-16s]	ThermoFisher Scientific	Cat# 48-5773-82; RRID: AB_1518812
Anti-human CD4 PerCP-Cy5.5 [SK3]	BD Biosciences	Cat# 566316; RRID: AB_2739678
Anti-mouse CD4 eFluor450 [RM4-5]	ThermoFisher Scientific	Cat# 48-0042-82; RRID: AB_1272194
Anti-mouse CD49b PE-Cy7 [DX5]	BioLegend	Cat# 108922; RRID: AB_2561460
Anti-mouse CD16/32 [2.4G2]	BD Biosciences	Cat# 553142; RRID: AB_394657
Anti-mouse FOXP3 APC [FJK-16s]	ThermoFisher Scientific	Cat# 17-5773-82; RRID: AB_469457
Anti-mouse CD8a BV570 [53-6.7]	BioLegend	Cat# 100739; RRID: AB_10897645
Anti-mouse Ki-67 AlexaFluor 488 [16A8]	BioLegend	Cat# 652417; RRID: AB_2564236
Anti-mouse CD44 PerCP-Cy5.5 [IM7]	ThermoFisher Scientific	Cat# 45-0441-82; RRID: AB_925746
Anti-mouse/human Helios PE/Dazzle 594 [22F6]	BioLegend	Cat# 137231; RRID: AB_2565796
Anti-mouse CD45 APC-Cy7 [QA17A26]	BioLegend	Cat# 157617; RRID: AB_2890720
Anti-mouse TER-119 APC-Cy7 [TER-119]	BioLegend	Cat# 116223; RRID: AB_2137788
Anti-human CD3 BV605[OKT3]	BioLegend	Cat# 317321; RRID: AB_11126166
Anti-human CD56 PE [TULY56]	ThermoFisher Scientific	Cat# 12-0566-41; RRID: AB_2572562
Anti-human CD4 PE-eFluor610 [RPA-T4]	ThermoFisher Scientific	Cat# 61-0049-42; RRID: AB_2574522
Anti-human CD8 BV711 [SK1]	BioLegend	Cat# 344733; RRID: AB_2565242
Anti-human IL-2Rα PE-Cy7 [M-A251]	BD Biosciences	Cat# 557741; RRID: AB_396847
Anti-human FOXP3 AlexaFluor 647 [259D]	BioLegend	Cat# 320214; RRID: AB_492984
Anti-human Ki-67 FITC [20Raj1]	ThermoFisher Scientific	Cat# 11-5699-42; RRID: AB_10687464
Anti-mouse CD45 PerCP-Cy5.5 [30-F11]	BioLegend	Cat# 103132; RRID: AB_893340
Anti-mouse CD3 BV605 [17A2]	BioLegend	Cat# 100237; RRID: AB_2562039
Anti-mouse CD4 APC [GK1.5]	BioLegend	Cat# 100411; RRID: AB_312696
Anti-mouse CD8a BV786 [53-6.7]	BD Biosciences	Cat# 563332; RRID: AB_2721167
Anti-mouse IL-2Rα BV421 [7D4]	BD Biosciences	Cat# 564571; RRID: AB_2738849
Anti-mouse FOXP3 FITC [FJK-16s]	ThermoFisher Scientific	Cat# 11-5773-82; RRID: AB_465243
Anti-mouse NK-1.1 PerCP-Cy5.5 [PK136]	BioLegend	Cat# 108727; RRID: AB_2132706
Anti-mouse CD45.1 APC-Cy7 [A20]	BioLegend	Cat# 110716; RRID: AB_313505
Anti-mouse CD45.2 PerCP-Cy5.5 [104]	BioLegend	Cat# 109828; RRID: AB_893350
Anti-mouse CD62L PE [MEL-14]	BioLegend	Cat# 104408; RRID: AB_313095
Anti-mouse CD44 AlexaFluor 700 [IM7]	BioLegend	Cat# 103026; RRID: AB_493713
Anti-mouse CD16/32 [2.4G2]	Bio X Cell	Cat# BP0307; RRID: AB_2736987
Rat IgG Isotype Control	ThermoFisher Scientific	Cat# 10700; RRID: AB_2610661
Anti-mouse CD4 BUV563 [GK1.5]	BD Biosciences	Cat# 612923; RRID: AB_2870208
Anti-mouse CD8a BUV615 [53-6.7]	BD Biosciences	Cat# 613004; RRID: AB_2870272
Anti-mouse CD11a BUV805 [2D7]	BD Biosciences	Cat# 741919; RRID: AB_2871232
Anti-mouse IL-2Rα BV785 [PC61]	BioLegend	Cat# 102051; RRID: AB_2564131
Anti-mouse CD27 BV650 [LG.3A10]	BioLegend	Cat# 124233; RRID: AB_2687192
Anti-mouse CD44 BV570 [IM7]	BioLegend	Cat# 103037; RRID: AB_10900641
Anti-mouse CD69 BUV737 [H1.2F3]	BD Biosciences	Cat# 612793; RRID: AB_2870120
Anti-mouse CD122 BUV661 [TM-β1]	BD Biosciences	Cat# 741493; RRID: AB_2870951
Anti-mouse KLRG1 BUV395 [2F1]	BD Biosciences	Cat# 740279; RRID: AB_2740018
Anti-mouse ICOS APC/Fire 750 [C398.4A]	BioLegend	Cat# 313536; RRID: AB_2632923
Anti-mouse PD-1 BV421 [29F.1A12]	BioLegend	Cat# 135218; RRID: AB_2561447
Anti-mouse BCL-2 AlexaFluor 647 [BCL/10C4]	BioLegend	Cat# 633510; RRID: AB_2274702
Anti-mouse CD3 BV570 [17A2]	BioLegend	Cat# 100249; RRID: AB_2734148
Anti-mouse CTLA-4 APC-R700 [UC10-4F10-11]	BD Biosciences	Cat# 565778; RRID: AB_2739350
Anti-mouse FOXP3 PE-Cy5.5 [FJK-16s]	ThermoFisher Scientific	Cat# 35-5773-82; RRID: AB_11218094
Anti-mouse Ki-67 AlexaFluor 488 [B56]	BD Biosciences	Cat# 558616; RRID: AB_647087
Anti-mouse T-bet PE-Cy5 [4B10]	ThermoFisher Scientific	Cat# 15-5825-82; RRID: AB_2815071
Anti-mouse CXCR3 BV650 [CXCR3-173]	BioLegend	Cat# 126531; RRID: AB_2563160
Anti-mouse NK-1.1 BV711 [PK136]	BioLegend	Cat# 108745; RRID: AB_2563286
Anti-mouse NKp46 BV605 [29A1.4]	BioLegend	Cat# 137619; RRID: AB_2562452
Anti-mouse PD-1 [RMP1-14]	Bio X Cell	Cat# BE0146; RRID: AB_10949053

Bacterial and virus strains		

One Shot™ MAX Efficiency™ DH5α-T1R Competent Cells	ThermoFisher Scientific	Cat# 12297016

Biological samples		

Human PBMCs	Anne Arundel Medical Blood Donor Center; NHS Blood and Transport	N/A

Chemicals, peptides, and recombinant proteins		

Polyethylenimine, Linear, MW 25000, Transfection Grade (PEI 25K™)	Polysciences	Cat# 23966
OptiPRO™ SFM	ThermoFisher Scientific	Cat# 12309019
FreeStyle™ 293 Expression Medium	ThermoFisher Scientific	Cat# 12338018
Pierce™ Protein G Agarose	ThermoFisher Scientific	Cat# 20397
F5111 IC LN15	This manuscript; see Table S1	N/A
F5111 IC LN25	This manuscript; see Table S1	N/A
F5111 IC LN35	This manuscript; see Table S1	N/A
F5111 IC LN35 (Y35A)	This manuscript; see Table S1	N/A
F5111 IC LN35 (Y52A)	This manuscript; see Table S1	N/A
F5111 IC LN35 (Y54A)	This manuscript; see Table S1	N/A
F5111 IC LN35 (Y60A)	This manuscript; see Table S1	N/A
F5111 IC LN35 (V103A)	This manuscript; see Table S1	N/A
F5111 IC LN35 (Y33A)	This manuscript; see Table S1	N/A
F5111 IC LN35 (Y94A)	This manuscript; see Table S1	N/A
F5111 IC LN35 (S96A)	This manuscript; see Table S1	N/A
F5111.2 IC LN35	This manuscript; see Table S1	N/A
Control (FITC-E2) IC LN35	This manuscript; see Table S1	N/A
F5111 IC LN35 (N297A)	This manuscript; see Table S1	N/A
F5111 IC LN35 (Y60A, N297A)	This manuscript; see Table S1	N/A
F5111 IC LN35 (V103A, N297A)	This manuscript; see Table S1	N/A
F5111 IC LN35 (Y33A, N297A)	This manuscript; see Table S1	N/A
F5111.2 IC LN35 (N297A)	This manuscript; see Table S1	N/A
Control (FITC-E2) IC LN35 (N297A)	This manuscript; see Table S1	N/A
Human IL-2	This manuscript	N/A
Biotinylated human IL-2	This manuscript	N/A
Biotinylated human IL-2Rα	This manuscript	N/A
Biotinylated human IL-2Rβ	This manuscript	N/A
Ni-NTA Agarose	Qiagen	Cat# 30210
16% Paraformaldehyde (formaldehyde) aqueous solution	Electron Microscopy Science	Cat# 15710
Ficoll® Paque Plus	MilliporeSigma	Cat# GE17-1440-02
ACK Lysing Buffer	Quality Biological	Cat# 118-156-101
LIVE/DEAD™ Fixable Blue Dead Cell Stain Kit	ThermoFisher Scientific	Cat# L34961
eBioscience™ Fixable Viability Dye eFluor™ 780	ThermoFisher Scientific	Cat# 65-0865-18
Violet Proliferation Dye 450	BD Biosciences	Cat# 562158
Zombie NIR™ Fixable Viability Kit	BioLegend	Cat# 423105
NHS-Rhodamine (5/6-carboxy-tetramethyl-rhodamine succinimidyl ester), mixed isomer	ThermoFisher Scientific	Cat# 46406
CD4 (L3T4) MicroBeads, mouse	Miltenyi	Cat# 130-117-043
CellTrace™ Violet Cell Proliferation Kit	ThermoFisher Scientific	Cat# C34571
Dynabeads™ Mouse T-Activator CD3/CD28 for T-Cell Expansion and Activation	ThermoFisher Scientific	Cat# 11452D
Ghost Dye™ Violet 510	Tonbo Biosciences	Cat# 13-0870-T100
Tetramer H2k(b) Tgd057 PE: SVLAFRRL	NIH Tetramer Core Facility; Wilson et al., 2010	N/A
Tetramer I-A(b) AS15 PE: AVEIHRPVPGTAPPS	NIH Tetramer Core Facility; Grover et al., 2012	N/A
Power SYBR™ Green PCR Master Mix	ThermoFisher Scientific	Cat# 4368577
Dextran sulfate sodium salt, colitis grade (36,000–50,000)	MP Biomedicals	Cat# 160110; Lot# S5036

Critical commercial assays		

BirA biotin-protein ligase standard reaction kit	Avidity	Cat# BirA500
Octet® Streptavidin (SA) Biosensor	Sartorius	Cat# 18-5019
Transcription Factor Phospho Buffer Set	BD Biosciences	Cat# 565575
eBioscience™ Foxp3/Transcription Factor Staining Buffer Set	ThermoFisher Scientific	Cat# 00-5523-00
DNeasy Blood & Tissue Kit	Qiagen	Cat# 69506

Experimental models: Cell lines		

FreeStyle™ 293-F Cells	ThermoFisher Scientific	Cat# R79007
YT-1 Human NK Cells	Yodoi et al., 1985	N/A
IL-2Rα^+^ YT-1 Human NK Cells	Kuziel et al., 1993	N/A

Experimental models: Organisms/strains		

Mouse: NOD/ShiLt	The Jackson Laboratory	Strain #:001976; RRID: IMSR_JAX:001976
Mouse: BALB/c Rag2^−/−^ γ_c_^−/−^ H2^d^ (BRG)	The Jackson Laboratory	Strain #: 014593 RRID: IMSR_JAX:014593
Mouse: C57BL/6	The Jackson Laboratory	Strain #:000664; RRID: IMSR_JAX:000664
Mouse: C57BL/6 CD45.1	The Jackson Laboratory	Strain #:002014; RRID: IMSR_JAX:002014
Mouse: C57BL/6 FOXP3-IRES-mRFP	The Jackson Laboratory	Strain #:008374; RRID: IMSR_JAX:008374
Mouse: C57BL/6 CD45.1; RFP-FOXP3 mice	This manuscript; CD45.1 mice were bred in house to FoxP3-RFP mice to homozygosity and maintained at Johns Hopkins University Cancer Research Building 1 use facility	N/A
Mouse: C57BL/6	Taconic Biosciences	Model: B6-M
*Toxoplasma gondii* – ME49 strain	N/A	N/A
Mouse: CBA/Ca	The Jackson Laboratory	Strain #: 000654 RRID: IMSR_JAX:000654
Mouse: BALB/c	Purchased from Charles River Laboratories and bred at Czech Centre for Phenogenomics	Strain: 028

Oligonucleotides		

qPCR primer: TCCCCTCTGCTGGCGAAAAGT (*Toxoplasma gondii* forward)	This manuscript; Lin et al., 2000	N/A
qPCR primer: AGCGTTCGTGGTCAACTATCGATTG (*Toxoplasma gondii* reverse)	This manuscript; Lin et al., 2000	N/A

Recombinant DNA		

Human IL-2 sequence	GenBank	GenBank: X00695.1
Mouse IL-2 sequence	GenBank	GenBank: X01772.1
pCT3CBN	Derived from pCT302 (Boder and Wittrup, 1997)	N/A
pCT3CBN_hIL2 (Yeast display vector for human IL-2)	This manuscript	N/A
pCT3CBN_mIL2 (Yeast display vector for mouse IL-2)	This manuscript	N/A
F5111 V_H_ and V_L_ sequence	Rondon et al., 2015	N/A
F5111.2 V_H_ and V_L_ sequence	Rondon et al., 2015	N/A
FITC-E2 V_H_ and V_L_ sequence	Honegger et al., 2005	N/A
Human IgG1 constant heavy chain sequence	ImMunoGeneTics (IMGT)	IMGT: J00228
Human IgG1 constant lambda chain sequence	ImMunoGeneTics (IMGT)	IMGT: J00253
Human IL-2Rα sequence (1–217)	GenBank	GenBank: X01057.1
Human IL-2Rβ sequence (1–214)	GenBank	GenBank: M26062.1
gWiz High Expression Blank Vector	Genlantis	P000200
gWiz_F5111_Ab_HC (Expression vector for F5111 heavy chain)	This manuscript	N/A
gWiz_F5111_Ab_LC (Expression vector for F5111 light chain)	This manuscript	N/A
gWiz_F5111.2_Ab_HC (Expression vector for F5111.2 heavy chain)	This manuscript	N/A
gWiz_F5111.2_Ab_LC (Expression vector for F5111.2 light chain)	This manuscript	N/A
gWiz_F5111_IC_LN15_LC (Expression vector for human IL-2 linked to F5111 light chain with a 15 amino acid linker)	This manuscript	N/A
gWiz_F5111_IC_LN25_LC (Expression vector for human IL-2 linked to F5111 light chain with a 25 amino acid linker)	This manuscript	N/A
gWiz_F5111_IC_LN35_LC (Expression vector for human IL-2 linked to F5111 light chain with a 35 amino acid linker)	This manuscript	N/A
gWiz_F5111_Ab_HC_Y35A (Expression vector for F5111 heavy chain with Y35A mutation)	This manuscript	N/A
gWiz_F5111_Ab_HC_Y52A (Expression vector for F5111 heavy chain with Y52A mutation)	This manuscript	N/A
gWiz_F5111_Ab_HC_Y54A (Expression vector for F5111 heavy chain with Y54A mutation)	This manuscript	N/A
gWiz_F5111_Ab_HC_Y60A (Expression vector for F5111 heavy chain with Y60A mutation)	This manuscript	N/A
gWiz_F5111_Ab_HC_V103A (Expression vector for F5111 heavy chain with V103A mutation)	This manuscript	N/A
gWiz_F5111_IC_LN35_LC_Y33A (Expression vector for human IL-2 linked to F5111 Y33A mutant light chain with a 35 amino acid linker)	This manuscript	N/A
gWiz_F5111_IC_LN35_LC_Y94A (Expression vector for human IL-2 linked to F5111 Y94A mutant light chain with a 35 amino acid linker)	This manuscript	N/A
gWiz_F5111_IC_LN35_LC_S96A (Expression vector for human IL-2 linked to F5111 S96A mutant light chain with a 35 amino acid linker)	This manuscript	N/A
gWiz_F5111.2_IC_LN35_LC (Expression vector for human IL-2 linked to F5111.2 light chain with a 35 amino acid linker)	This manuscript	N/A
gWiz_Control_IC_HC (Expression vector for FITC-E2 heavy chain)	This manuscript	N/A
gWiz_Control_IC_LN35_LC (Expression vector for human IL-2 linked to FITC-E2 light chain with 35 amino acid linker)	This manuscript	N/A
gWiz_F5111_Ab_HC_N297A (Expression vector for F5111 heavy chain with N297A mutation)	This manuscript	N/A
gWiz_F5111.2_Ab_HC (Expression vector for F5111.2 heavy chain with N297A mutation)	This manuscript	N/A
gWiz_F5111_Ab_HC_Y60A_N297A (Expression vector for F5111 heavy chain with Y60A and N297A mutations)	This manuscript	N/A
gWiz_F5111_Ab_HC_V103A_N297A (Expression vector for F5111 heavy chain with V103A and N297A mutations)	This manuscript	N/A
gWiz_Control_IC_HC_N297A (Expression vector for FITC-E2 heavy chain with N297A)	This manuscript	N/A
gWiz_hIL2 (Expression vector for human IL-2)	This manuscript	N/A
gWiz_hIL2_BH3 (Expression vector for human IL-2 with BH3 tag for biotinylation)	This manuscript	N/A
gWiz_hIL2Ra_BH3 (Expression vector for human IL-2Rα residues 1–217 with BH3 tag for biotinylation)	This manuscript	N/A
gWiz_hIL2Rb_BH3 (Expression vector for human IL-2Rβ residues 1–214 with BH3 tag for biotinylation)	This manuscript	N/A
gWiz_Trastuzumab_HC (Expression vector for trastuzumab heavy chain)	This manuscript	N/A
gWiz_Trastuzumab_LC (Expression vector for trastuzumab light chain)	This manuscript	N/A

Software and algorithms		

GraphPad Prism 8.4.3	GraphPad	https://www.graphpad.com/
FlowJo v10.7.1	FlowJo, LLC	https://www.flowjo.com/solutions/flowjo
PyMOL v2.3.2	PyMOL	https://pymol.org/2/
BioRender	BioRender	https://biorender.com/

## Highlights

- Single-chain human interleukin-2/antibody fusion protein (immunocytokine) is designed
- F5111 immunocytokine promotes biased Treg activation and expansion
- Immunocytokine induces superior Treg bias compared with cytokine/antibody complex
- F5111 immunocytokine confers protection in mouse models of autoimmune diseases
