# Supplementary material for: Engineered human cytokine/antibody fusion proteins expand regulatory T cells and confer autoimmune disease protection
Source: Cell Rep. Author manuscript; Available in PMC 2022 Nov 3. (PMC9631798; doi:10.1016/j.celrep.2022.111478)
Supplement: 1 [file NIHMS1843698-supplement-1.pdf]

Cell Reports, Volume *41*

Supplemental information

Engineered human cytokine/antibody fusion proteins expand regulatory T cells and confer autoimmune disease protection

Derek VanDyke, Marcos Iglesias, Jakub Tomala, Arabella Young, Jennifer Smith, Joseph A. Perry, Edward Gebara, Amy R. Cross, Laurene S. Cheung, Arbor G. Dykema, Brian T. Orcutt-Jahns, Tereza Henclová, Jaroslav Golias, Jared Balolong, Luke M. Tomasovic, David Funda, Aaron S. Meyer, Drew M. Pardoll, Joanna Hester, Fadi Issa, Christopher A. Hunter, Mark S. Anderson, Jeffrey A. Bluestone, Giorgio Raimondi, and Jamie B. Spangler

Figure S1

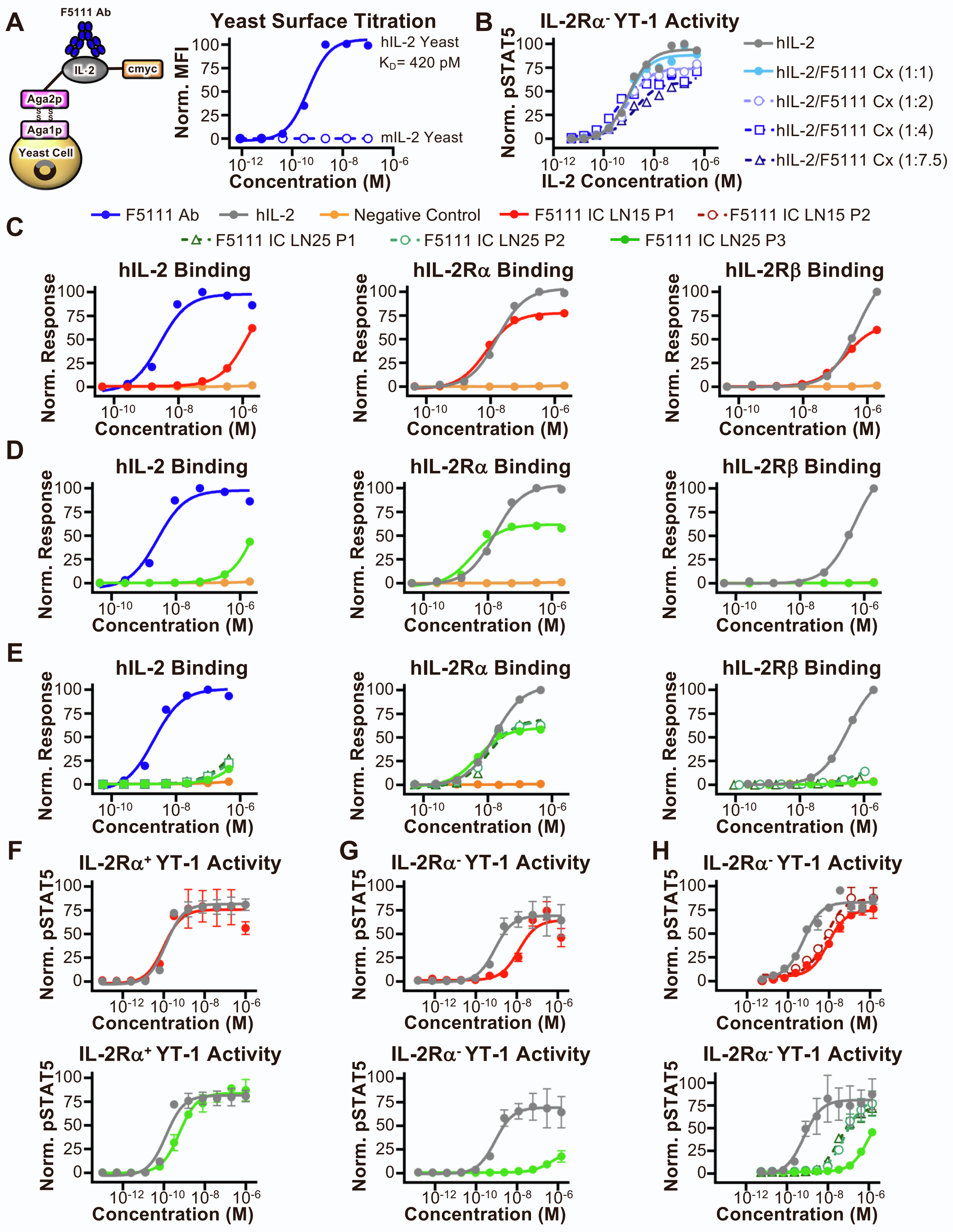

A
F5111 Ab
IL-2
cmyc
Aga2p
Aga1p
Yeast Cell
Yeast Surface Titration
hIL-2 Yeast
K_D= 420 pM
mIL-2 Yeast
Norm. MFI
Concentration (M)
B
IL-2Rα- YT-1 Activity
Norm. pSTAT5
IL-2 Concentration (M)
hIL-2
hIL-2/F5111 Cx (1:1)
hIL-2/F5111 Cx (1:2)
hIL-2/F5111 Cx (1:4)
hIL-2/F5111 Cx (1:7.5)
C
F5111 Ab
hIL-2
Negative Control
F5111 IC LN15 P1
F5111 IC LN15 P2
F5111 IC LN25 P1
F5111 IC LN25 P2
F5111 IC LN25 P3
hIL-2 Binding
hIL-2Rα Binding
hIL-2Rβ Binding
Norm. Response
Concentration (M)
D
E
F
IL-2Rα+ YT-1 Activity
G
IL-2Rα- YT-1 Activity
H
Norm. pSTAT5

Figure S1. Increasing IC linker length improves Treg bias. Related to Figures 1 and 2. (A) Binding of the F5111 antibody (Ab) to yeast-displayed hIL-2 or mIL-2. Fitted equilibrium dissociation constant (K_D) is shown. **(B)** STAT5 phosphorylation response of IL-2Rα^- YT-1 human NK cells stimulated with either hIL-2 or hIL-2/F5111 complex (Cx) at varying molar ratios of cytokine to antibody. **(C-E)** Equilibrium biolayer interferometry-based titrations of hIL-2, F5111 antibody (Ab), Negative Control (trastuzumab), F5111 IC LN15 P1, and F5111 IC LN25 P1, P2, and P3 binding to immobilized hIL-2 (left), immobilized hIL-2Rα (middle), and immobilized hIL-2Rβ (right). **(F)** STAT5 phosphorylation response of IL-2Rα^+ YT-1 human NK cells stimulated with either hIL-2, F5111 IC LN15 P1 (top), or F5111 IC LN25 P3 (bottom). **(G)** STAT5 phosphorylation response of IL-2Rα^- YT-1 human NK cells stimulated with either hIL-2, F5111 IC LN15 P1 (top), or F5111 IC LN25 P3 (bottom). **(H)** STAT5 phosphorylation response of IL-2Rα^- YT-1 human NK cells stimulated with either hIL-2, F5111 IC LN15 P1 and P2 (top), or F5111 IC LN25 P1, P2, and P3 (bottom). Data in **(F-H)** represent mean ± SD (n=3). See also **Tables S2 and S3**.

Figure S2

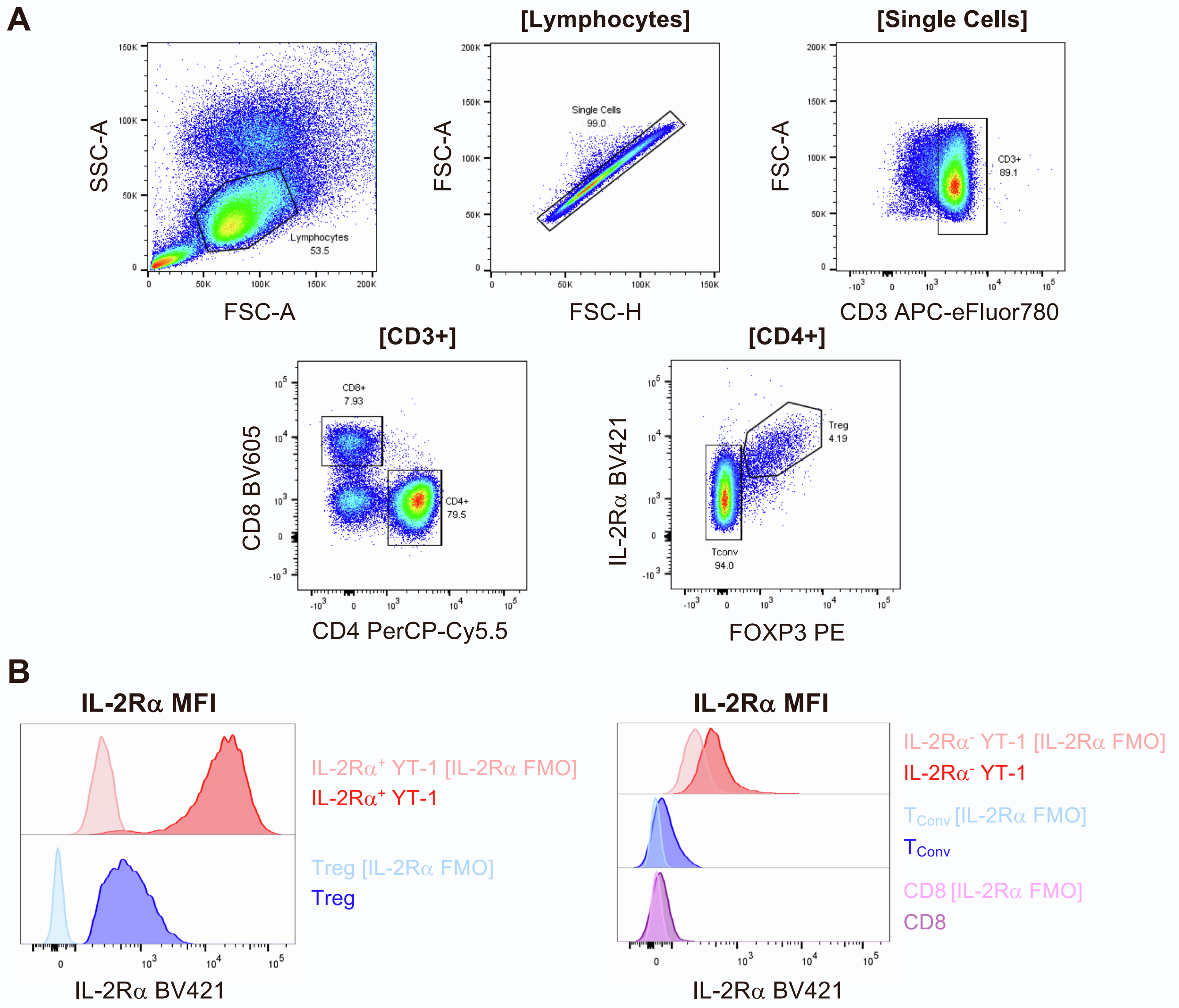

Figure S2. IL-2Rα$^+$ YT-1 cells express higher levels of IL-2Rα than human Tregs. Related to Figures 2, 3, and 5. (A) Representative flow cytometry plots illustrating the gating strategy used for human PBMCs. **(B)** Representative histograms illustrating IL-2Rα MFI of IL-2Rα$^+$ YT-1 cells and human Tregs (left) and IL-2Rα$^-$ YT-1 cells, human CD8$^+$ T cells, and human T_{Conv} cells (right) as compared to the IL-2Rα fluorescence minus one (FMO) controls.

Figure S3

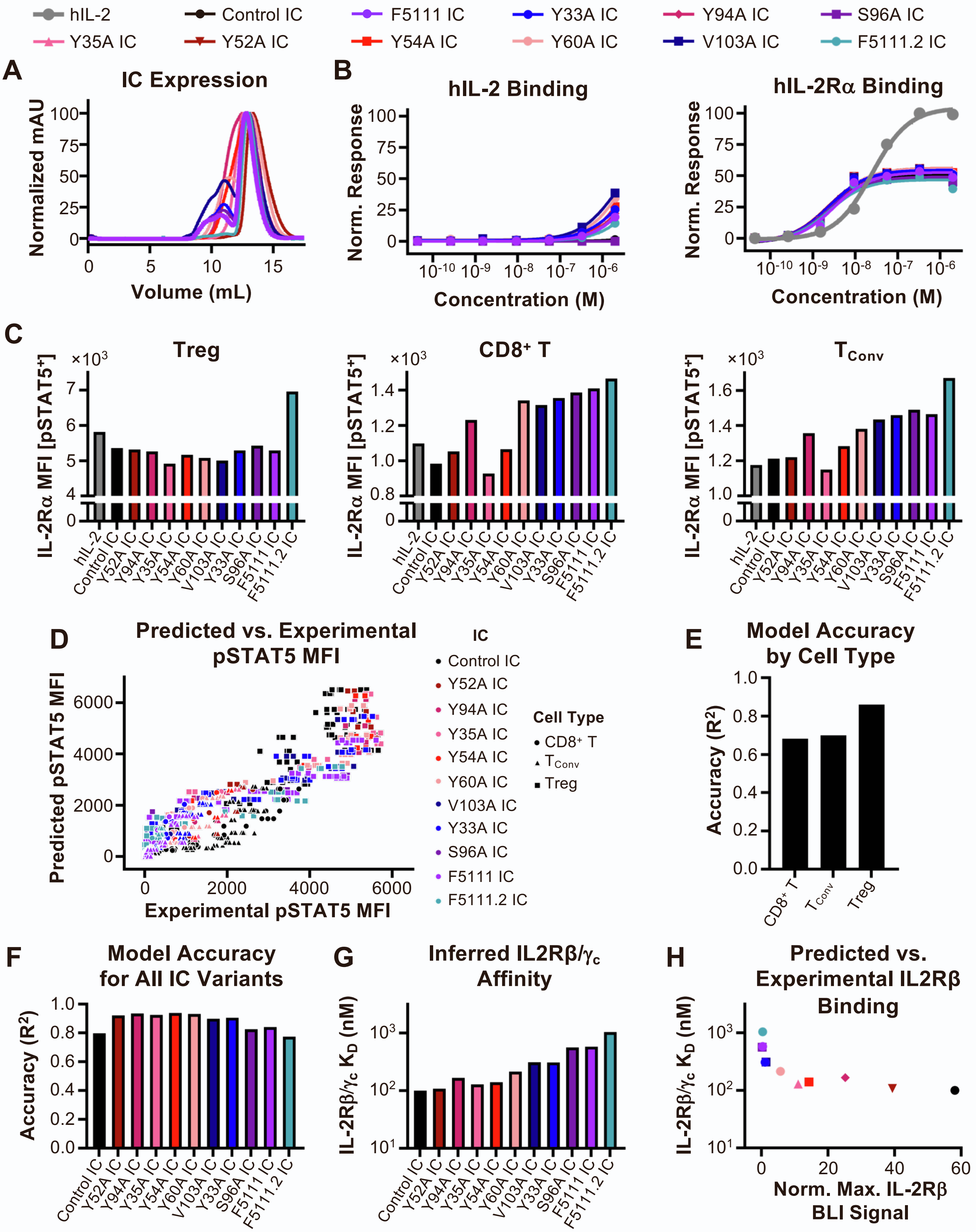

hIL-2
Control IC
F5111 IC
Y33A IC
Y94A IC
S96A IC
Y35A IC
Y52A IC
Y54A IC
Y60A IC
V103A IC
F5111.2 IC
A
IC Expression
Normalized mAU
Volume (mL)
B
hIL-2 Binding
hIL-2Rα Binding
Norm. Response
Concentration (M)
C
Treg
CD8+ T
TConv
IL-2Rα MFI [pSTAT5+]
×10³
D
Predicted vs. Experimental pSTAT5 MFI
Predicted pSTAT5 MFI
Experimental pSTAT5 MFI
IC
Cell Type
CD8+ T
TConv
Treg
E
Model Accuracy by Cell Type
Accuracy (R²)
F
Model Accuracy for All IC Variants
Accuracy (R²)
G
Inferred IL2Rβ/γc Affinity
IL-2Rβ/γc KD (nM)
H
Predicted vs. Experimental IL2Rβ Binding
IL-2Rβ/γc KD (nM)
Norm. Max. IL-2Rβ BLI Signal

Figure S3. Characterization of F5111 IC variants. Related to Figures 3 and 4. (A) Overlay of SEC traces for F5111 IC variants. **(B)** Equilibrium biolayer interferometry-based titrations of hIL-2, Control IC, and F5111 IC variants binding to immobilized hIL-2 (left) and immobilized hIL-2Rα (right). Binding to immobilized hIL-2 was normalized based on the binding of the F5111.2 antibody (**Figure S5C, left**). **(C)** IL-2Rα MFI within the pSTAT5$^+$ population of Treg (left), CD8$^+$ T (middle), and T_{Conv} (right) cell populations within human PBMCs stimulated with either hIL-2, Control IC, or F5111 IC variants at treatment concentrations of 2 nM (left) and 200 nM (middle and right). **(D)** Predicted vs. experimentally measured pSTAT5 MFI for all cell types, ICs, and concentrations modeled. Each point represents a single experimental measurement (n=1). **(E)** Model accuracy delineated by cell type for all ICs. **(F)** Model accuracy delineated by treatment for all ICs. Accuracies are calculated as a Pearson's correlation R^2. **(G)** Inferred IL2Rβ/γ_c equilibrium dissociation constants (K_D, nM) for each IC. **(H)** Inferred IL2Rβ/γ_c equilibrium dissociation constants (K_D, nM) compared to the maximum normalized IL2Rβ biolayer interferometry (BLI) signal experimentally measured for each IC. See also **Table S2**.

Figure S4

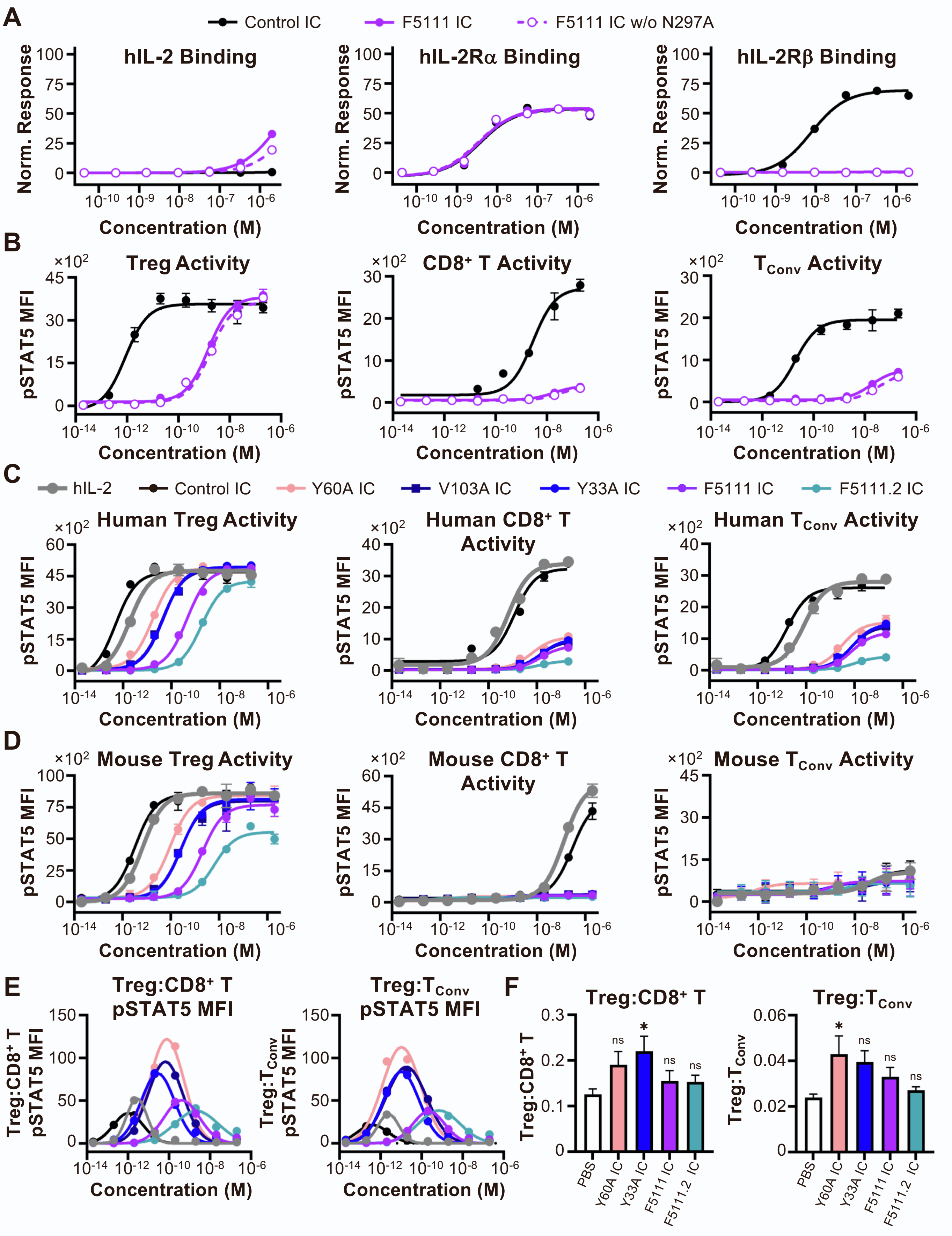
A
Control IC
F5111 IC
F5111 IC w/o N297A
hIL-2 Binding
hIL-2Rα Binding
hIL-2Rβ Binding
Norm. Response
Concentration (M)
B
Treg Activity
CD8+ T Activity
TConv Activity
pSTAT5 MFI
×10^2
C
hIL-2
Control IC
Y60A IC
V103A IC
Y33A IC
F5111 IC
F5111.2 IC
Human Treg Activity
Human CD8+ T Activity
Human TConv Activity
D
Mouse Treg Activity
Mouse CD8+ T Activity
Mouse TConv Activity
E
Treg:CD8+ T pSTAT5 MFI
Treg:TConv pSTAT5 MFI
F
Treg:CD8+ T
Treg:TConv
ns
*
PBS
Y60A IC
Y33A IC
F5111 IC
F5111.2 IC

Figure S4. The N297A mutation does not impact *in vitro* function and IC variants modulate Treg bias *in vitro* and *in vivo*. Related to Figures 3 and 4. (A) Equilibrium biolayer interferometry-based titrations of Control IC, F5111 IC, and F5111 IC without the N297A mutation (w/o N297A, intact effector function) binding to immobilized hIL-2 (left), immobilized hIL-2Rα (middle), and immobilized hIL-2Rβ (right). Binding to immobilized hIL-2 was normalized based on the binding of the F5111.2 antibody (**Figure S5C, left**). **(B)** STAT5 phosphorylation response of Treg (left), $CD8^+$ T (middle), and T_{Conv} (right) cell populations from human PBMCs stimulated with either Control IC, F5111 IC, or F5111 IC w/o N297A. Data represent mean ± SD (n=3). **(C)** STAT5 phosphorylation response of Treg (left), $CD8^+$ T (middle), and T_{Conv} (right) cell populations within human PBMCs stimulated with either hIL-2, Control IC, Y60A IC, V103A IC, Y33A IC, F5111 IC, or F5111.2 IC (all with N297A mutation). Data represent mean ± SD (n=3). **(D)** STAT5 phosphorylation response of Treg (left), $CD8^+$ T (middle), and T_{Conv} (right) cells isolated from spleens of NOD mice and stimulated with either hIL-2, Control IC, Y60A IC, V103A IC, Y33A IC, F5111 IC, or F5111.2 IC (all with N297A mutation). Data represent mean ± SD (n=3). **(E)** Average pSTAT5 MFI ratio of human Treg:$CD8^+$ T (left) and human Treg:T_{Conv} (right) for each IC determined at each stimulation concentration from the experiment shown in **(C)**. **(F)** BRG mice were administered 5×10^6 human PBMCs (*i.p.*) on day 0 and then on day 1 were treated (*i.p.*) with either PBS (n=6) or 8.2 µg (1.5 µg IL-2 equivalence) Y60A IC (n=5), Y33A IC (n=4), F5111 IC (n=4), or F5111.2 IC (n=5). Cells were collected by lavage from the peritoneum on day 4 and ratios of human Treg:$CD8^+$ T (left) and Treg:T_{Conv} (right) were evaluated. Data represent mean + SEM. Statistical significance was determined by one-way ANOVA with a Tukey post hoc test. Only statistical significance compared to PBS is shown on the plots. All statistical data are provided in **Table S6**. *P≤0.05, **P≤0.01, ***P≤0.001, ****P≤0.0001. See also **Tables S2, S4, and S5**.

Figure S5

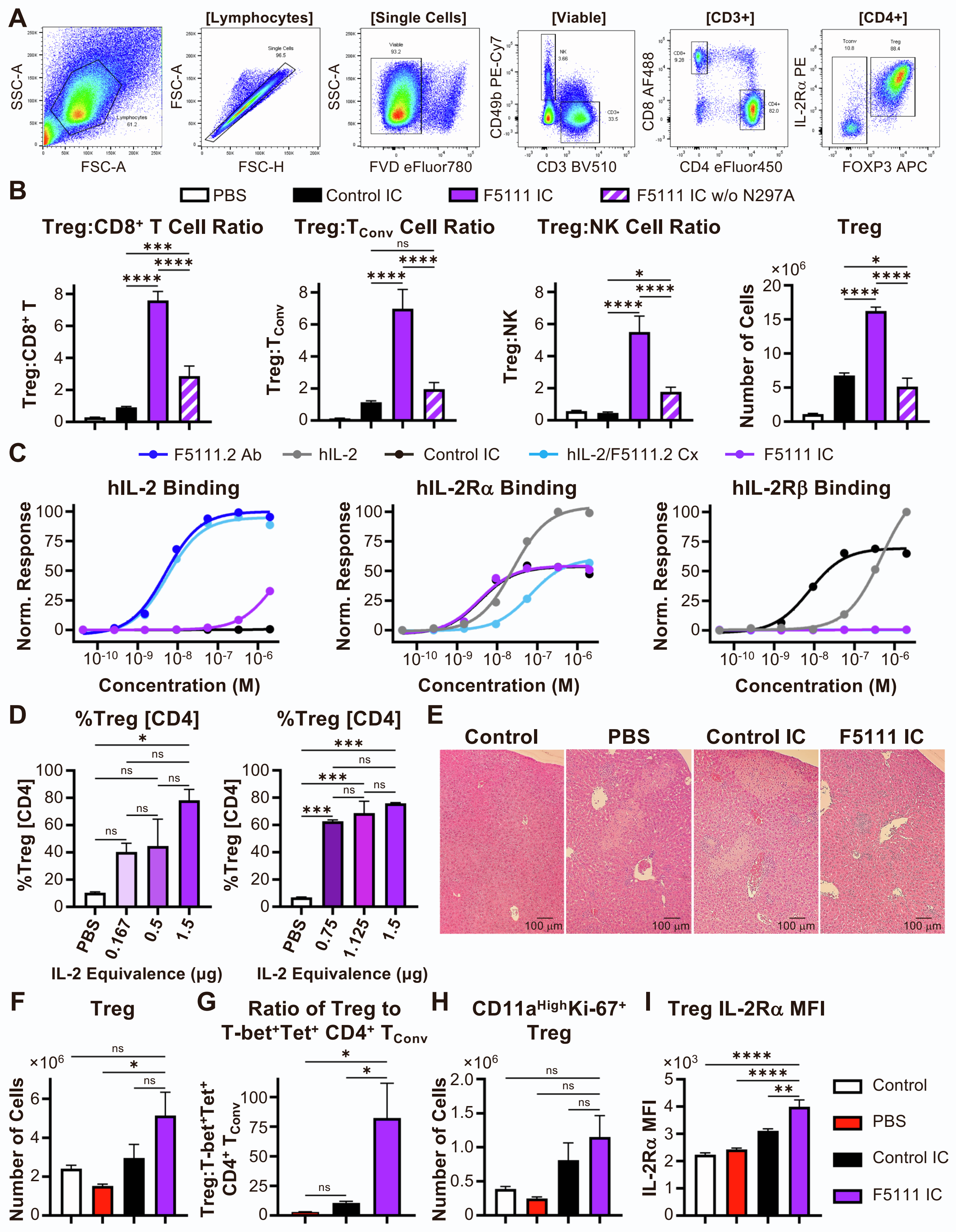

A
[Lymphocytes]
[Single Cells]
[Viable]
[CD3+]
[CD4+]
SSC-A
FSC-A
FSC-H
FVD eFluor780
CD49b PE-Cy7
CD3 BV510
CD8 AF488
CD4 eFluor450
IL-2Rα PE
FOXP3 APC
B
PBS
Control IC
F5111 IC
F5111 IC w/o N297A
Treg:CD8⁺ T Cell Ratio
Treg:T Conv Cell Ratio
Treg:NK Cell Ratio
Treg
Treg:CD8⁺ T
Treg:T Conv
Treg:NK
Number of Cells
C
F5111.2 Ab
hIL-2
Control IC
hIL-2/F5111.2 Cx
F5111 IC
hIL-2 Binding
hIL-2Rα Binding
hIL-2Rβ Binding
Norm. Response
Concentration (M)
D
%Treg [CD4]
IL-2 Equivalence (μg)
E
Control
PBS
Control IC
F5111 IC
100 μm
F
Treg
G
Ratio of Treg to T-bet⁺Tet⁺ CD4⁺ T Conv
Treg:T-bet⁺Tet⁺ CD4⁺ T Conv
H
CD11a^High Ki-67⁺ Treg
I
Treg IL-2Rα MFI
IL-2Rα MFI
Control
PBS
Control IC
F5111 IC

Figure S5. ***In vivo* characterization of F5111 IC. Related to Figures 4-6. (A)** Representative flow cytometry plots illustrating the gating strategy used for NOD mouse immune cell subset expansion studies. **(B)** Ratios of Treg to $CD8^+$ T cells (left), Treg to T_{Conv} cells (middle), and Treg to NK cells (right) in spleens harvested from NOD mice (n=4 per group) treated daily for four days (*i.p.*) with either PBS or 8.2 µg (1.5 µg IL-2 equivalence) Control IC, F5111 IC, or F5111 IC without the N297A mutation (w/o N297A, intact effector function). Data represent mean + SD. Statistical significance was determined by one-way ANOVA with a Tukey post hoc test. Significance is shown between Control IC and F5111 IC with and without the N297A mutation. **(C)** Equilibrium biolayer interferometry-based titrations of F5111.2 antibody (Ab), hIL-2, Control IC, hIL-2/F5111.2 complex (Cx, 1:1 molar ratio), and F5111 IC binding to immobilized hIL-2 (left), immobilized hIL-2Rα (middle), and immobilized hIL-2Rβ (right). **(D)** C57BL/6 mice (n=2 per group) were treated daily for 4 days (*i.p.*) with either PBS or varying dosages of F5111 IC: 0.91 µg (0.167 µg IL-2 equivalence); 2.7 µg (0.5 µg IL-2 equivalence); 4.1 µg (0.75 µg IL-2 equivalence); 6.2 µg (1.125 µg IL-2 equivalence); or 8.2 µg (1.5 µg IL-2 equivalence). Spleens were harvested 24 hours after the last dose. Percent of Tregs within the $CD4^+$ T cell population is shown. Data represent mean + SD. Statistical significance was determined by one-way ANOVA with a Tukey post hoc test. **(E-I)** C57BL/6 mice were administered (*i.p.*) 25 cysts of the ME-49 strain of *Toxoplasma gondii* (*T. gondii*) on day 0. Control group designates disease-free mice that were not given cysts. Starting on day 1, mice were treated daily for 5 days (*i.p.*) with either PBS (Control, n=5; PBS, n=4) or 8.2 µg (1.5 µg IL-2 equivalence) Control IC (n=5) or F5111 IC (n=5). Mice were sacrificed on day 10. **(E)** Representative H&E staining of harvested mouse livers. Scale bar, 100 µm. **(F)** Total number of Tregs in harvested spleen. **(G)** Ratio of Tregs to T-bet^+Tetramer $(\text{Tet})^+$ $CD4^+$ T_{Conv} cells in harvested mouse spleen. **(H)** Total number of $CD11a^{High}$$\text{Ki-67}^+$ Tregs in harvested mouse spleen. **(I)** IL-2Rα MFI of Tregs in harvested mouse spleen. Data in **(F-I)** represent mean + SEM. Statistical significance in **(F-I)** was determined by one-way ANOVA with a Tukey post hoc test. Significance compared to F5111 IC is shown. All statistical data are provided in **Table S6**. *P≤0.05, **P≤0.01, ***P≤0.001, ****P≤0.0001. See also **Table S2**.

Figure S6

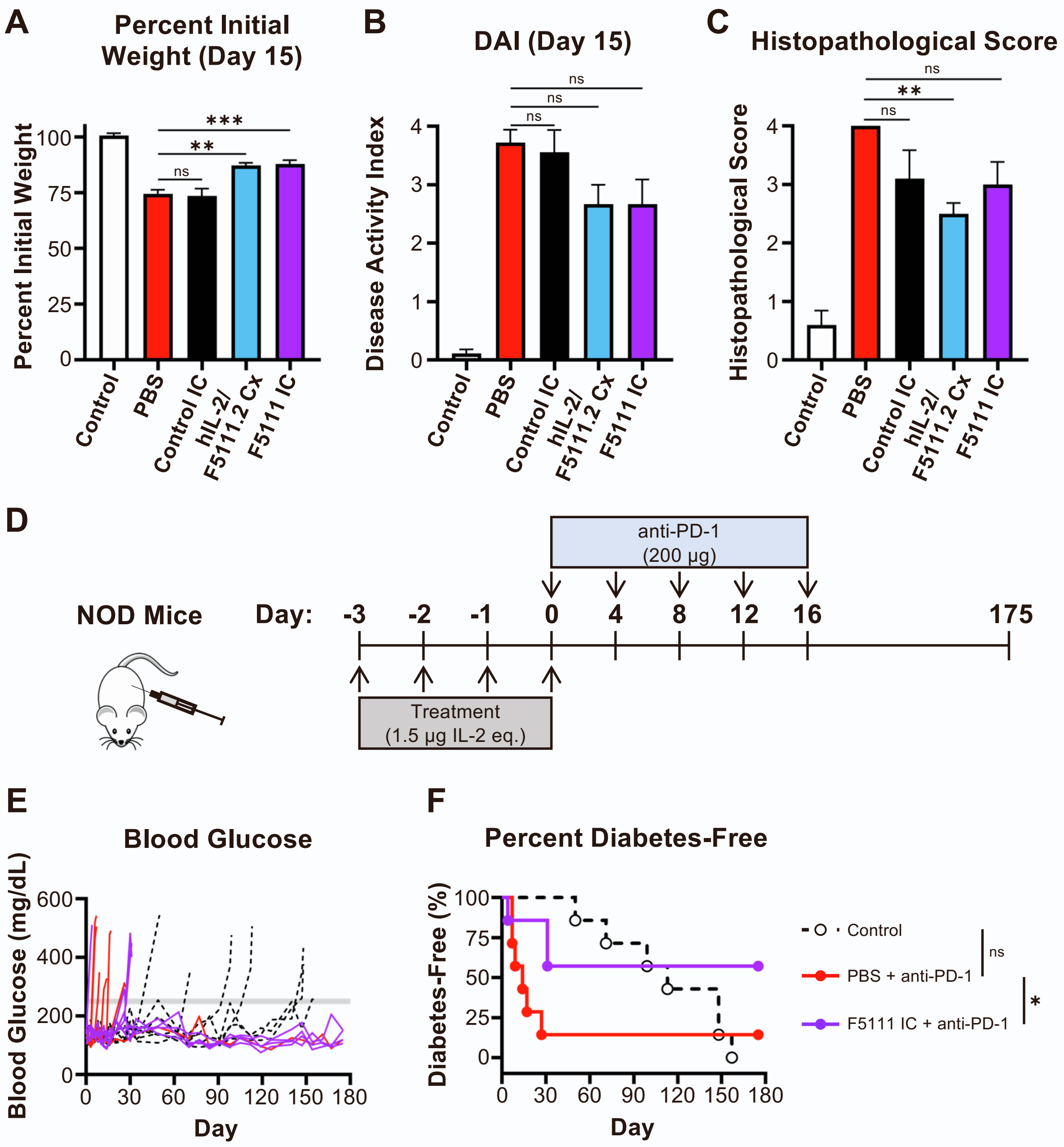
A
Percent Initial Weight (Day 15)
Percent Initial Weight
100
75
50
25
0
ns
**
***
Control
PBS
Control IC
hIL-2/ F5111.2 Cx
F5111 IC
B
DAI (Day 15)
Disease Activity Index
4
3
2
1
0
ns
ns
ns
Control
PBS
Control IC
hIL-2/ F5111.2 Cx
F5111 IC
C
Histopathological Score
Histopathological Score
4
3
2
1
0
ns
**
ns
Control
PBS
Control IC
hIL-2/ F5111.2 Cx
F5111 IC
D
anti-PD-1
(200 μg)
NOD Mice
Day:
-3
-2
-1
0
4
8
12
16
175
Treatment
(1.5 μg IL-2 eq.)
E
Blood Glucose
Blood Glucose (mg/dL)
600
400
200
0
0
30
60
90
120
150
180
Day
F
Percent Diabetes-Free
Diabetes-Free (%)
100
75
50
25
0
0
30
60
90
120
150
180
Day
Control
PBS + anti-PD-1
F5111 IC + anti-PD-1
ns
*

Figure S6. Evaluation of F5111 IC in mouse models of autoimmune disease. Related to Figure 7. (A-C) BALB/c mice (n=6 per group) were treated daily for 7 days (*i.p.*) with either PBS (Control and PBS), 1.5 μg hIL-2 complexed with 6.6 μg F5111.2 antibody (1:2 molar ratio, hIL-2/F5111.2 Cx), or 8.2 μg (1.5 μg IL-2 equivalence) Control IC or F5111 IC. Beginning on day 7, all groups except for the disease-free cohort (Control) were administered 3% DSS in their drinking water. Mice were sacrificed on day 15. **(A)** Weight change on day 15. **(B)** Disease activity index (DAI) on day 15. **(C)** Histopathology scores for H&E stained colons (n=5 Control, Control IC; n=6 PBS, Cx, F5111 IC). Data represent mean ± SEM. Statistical significance was determined by one-way ANOVA with a Tukey post hoc test. All plots show significance of Control IC, Cx, and F5111 IC treated mice versus PBS treated mice. **(D-F)** 8-week-old NOD mice (n=7 per group) were treated daily for 4 days (*i.p.*, days -3, -2, -1, 0) with either PBS (Control and PBS) or 8.2 μg (1.5 μg IL-2 equivalence) F5111 IC. Starting on day 0 (4 hours after the last IC dose), mice were administered anti-PD-1 antibody (200 μg) every 4 days until day 16. Control group designates mice that did not receive anti-PD-1 antibody. **(E)** Blood glucose concentrations over the study. The threshold 250 mg/dL value is indicated by the gray line. **(F)** Percent diabetes-free mice. Statistical significance was determined by pairwise comparisons using the Log-rank (Mantel-Cox) test. Statistical significance compared to mice treated with PBS + anti-PD-1 is shown. All statistical data are provided in **Table S6**. *P≤0.05, **P≤0.01, ***P≤0.001, ****P≤0.0001.

Table S1. Antibody and IC sequences. Related to Figures 1 and 3.

Construct	Amino Acid Sequence<br>Signal sequence – V_H or V_L – human IgG1 C_H1, C_H2, and C_H3 – hIL-2 – Linker – human Lambda C_L
**F5111 Heavy Chain**<br><br>(Single-point alanine mutations)<br><br>(N297A)	METDTLLLWVLLLWVPGSTGDQLQLQESGPGLVKPSQTLSLTCTVSGGSISSGGYYWSWIR<br>QHPGKGLEWIGYIYYSGSTYYNPSLKSRVTISVDTSKNQFSLKLSSVTAADTAVYYCARTPTV<br>TGDWFDPWGRGTLVTVSSASTKGPSVFPLAPSSKSTSGGTAALGCLVKDYFPEPVTVSWN<br>SGALTSGVHTFPAVLQSSGLYSLSSVVTVPSSSLGTQTYICNVNHKPSNTKVDKKVEPKSCD<br>KTHTCPPCPAPELLGGPSVFLFPPKPKDTLMISRTPEVTCVVVDVSHEDPEVKFNWYVDGVE<br>VHNAKTKPREEQYNSTYRVVSVLTVLHQDWLNGKEYKCKVSNKALPAPIEKTISKAKGQPRE<br>PQVYTLPPSREEMTKNQVSLTCLVKGFYPSDIAVEWESNGQPENNYKTTPPVLDSDGSFFL<br>YSKLTVDKSRWQQGNVFSCSVMHEALHNHYTQKSLSLSPGK
**F5111 Light Chain**	MRVPAQLLGLLLLWLPGARCGSNFMLTQPHSVSESPGKTVTISCTRSSGSIASNYVQWYQQ<br>RPGSSPTTVIYEDNQRPSGVPDRFSGSIDSSSNSASLTISGLKTEDEADYYCQSYDSSNVVF<br>GGGTKLTVLGQPKAAPSVTLFPPSSEELQANKATLVCLISDFYPGAVTVAWKADSSPVKAGV<br>ETTTPSKQSNNKYAASSYLSLTPEQWKSHRSYSCQVTHEGSTVEKTVAPTECS
**F5111 Light Chain + hIL-2**<br><br>**LN15:** X = 3<br>**LN25:** X = 5<br>**LN35:** X = 7<br><br>(Single-point alanine mutations)	MYRMQLLSCIALSLALVTNSAPTSSSTKKTQLQLEHLLLDLQMILNGINNYKNPKLTRMLTFKF<br>YMPKKATELKHLQCLEEELKPLEEVLNLAQSKNFHLRPRDLISNINVIVLELKGSETTFMCEYA<br>DETATIVEFLNRWITFCQSIISTLT$(GGGGS)_X$NFMLTQPHSVSESPGKTVTISCTRSSGSIASN<br>YVQWYQQRPGSSPTTVIYEDNQRPSGVPDRFSGSIDSSSNSASLTISGLKTEDEADYYCQS<br>YDSSNVVFGGGTKLTVLGQPKAAPSVTLFPPSSEELQANKATLVCLISDFYPGAVTVAWKAD<br>SSPVKAGVETTTPSKQSNNKYAASSYLSLTPEQWKSHRSYSCQVTHEGSTVEKTVAPTECS
**Control IC Heavy Chain**<br><br>(N297A)	METDTLLLWVLLLWVPGSTGDQVQLVESGGNLVQPGGSLRLSCAASGFTFGSFSMSWVRQ<br>APGGGLEWVAGLSARSSLTHYADSVKGRFTISRDNAKNSVYLQMNSLRVEDTAVYYCARRS<br>YDSSGYWGHFYSYMDVWGQGTLVTVSASTKGPSVFPLAPSSKSTSGGTAALGCLVKDYFP<br>EPVTVSWNSGALTSGVHTFPAVLQSSGLYSLSSVVTVPSSSLGTQTYICNVNHKPSNTKVDK<br>KVEPKSCDKTHTCPPCPAPELLGGPSVFLFPPKPKDTLMISRTPEVTCVVVDVSHEDPEVKF<br>NWYVDGVEVHNAKTKPREEQYNSTYRVVSVLTVLHQDWLNGKEYKCKVSNKALPAPIEKTI<br>SKAKGQPREPQVYTLPPSREEMTKNQVSLTCLVKGFYPSDIAVEWESNGQPENNYKTTPPV<br>LDSDGSFFLYSKLTVDKSRWQQGNVFSCSVMHEALHNHYTQKSLSLSPGK
**Control IC Light Chain + hIL-2**	MYRMQLLSCIALSLALVTNSAPTSSSTKKTQLQLEHLLLDLQMILNGINNYKNPKLTRMLTFKF<br>YMPKKATELKHLQCLEEELKPLEEVLNLAQSKNFHLRPRDLISNINVIVLELKGSETTFMCEYA<br>DETATIVEFLNRWITFCQSIISTLTGGGGSGGGGSGGGGSGGGGSGGGGSGGGGSGGGG<br>SSVLTQPSSVSAAPGQKVTISCSGSTSNIGNNYVSWYQQHPGKAPKLMIYDVSKRPSGVPD<br>RFSGSKSGNSASLDISGLQSEDEADYYCAAWDDSLSEFLFGTGTKLTVLGGQPKAAPSVTLF<br>PPSSEELQANKATLVCLISDFYPGAVTVAWKADSSPVKAGVETTTPSKQSNNKYAASSYLSL<br>TPEQWKSHRSYSCQVTHEGSTVEKTVAPTECS
**F5111.2 Heavy Chain**<br><br>(N297A)	METDTLLLWVLLLWVPGSTGDQLQLQESGPGLVKPSQTLSLTCTVSGGSISSGGYYWSWIR<br>QHPGKGLEWIGYIYKSGSAYYSPSLKSRVTISVDTSKNQFSLKLSSVTAADTAVYYCARTPTV<br>TGDWFDPWGRGTLVTVSSASTKGPSVFPLAPSSKSTSGGTAALGCLVKDYFPEPVTVSWN<br>SGALTSGVHTFPAVLQSSGLYSLSSVVTVPSSSLGTQTYICNVNHKPSNTKVDKKVEPKSCD<br>KTHTCPPCPAPELLGGPSVFLFPPKPKDTLMISRTPEVTCVVVDVSHEDPEVKFNWYVDGVE<br>VHNAKTKPREEQYNSTYRVVSVLTVLHQDWLNGKEYKCKVSNKALPAPIEKTISKAKGQPRE<br>PQVYTLPPSREEMTKNQVSLTCLVKGFYPSDIAVEWESNGQPENNYKTTPPVLDSDGSFFL<br>YSKLTVDKSRWQQGNVFSCSVMHEALHNHYTQKSLSLSPGK
**F5111.2 Light Chain**	MRVPAQLLGLLLLWLPGARCGSNFMLTQPHSVSESPGKTVTISCTRSSGSIASNYVQWYQQ<br>RPGSSPTTVIYEDNQRPSGVPDRFSGSIDSSSNSASLTISGLKTEDEADYYCQTYDSIDVYFG<br>GGTKLTVLGQPKAAPSVTLFPPSSEELQANKATLVCLISDFYPGAVTVAWKADSSPVKAGVE<br>TTTPSKQSNNKYAASSYLSLTPEQWKSHRSYSCQVTHEGSTVEKTVAPTECS
**F5111.2 Light Chain + hIL-2**	MYRMQLLSCIALSLALVTNSAPTSSSTKKTQLQLEHLLLDLQMILNGINNYKNPKLTRMLTFKF<br>YMPKKATELKHLQCLEEELKPLEEVLNLAQSKNFHLRPRDLISNINVIVLELKGSETTFMCEYA<br>DETATIVEFLNRWITFCQSIISTLTGGGGSGGGGSGGGGSGGGGSGGGGSGGGGSGGGG<br>SNFMLTQPHSVSESPGKTVTISCTRSSGSIASNYVQWYQQRPGSSPTTVIYEDNQRPSGVP<br>DRFSGSIDSSSNSASLTISGLKTEDEADYYCQTYDSIDVYFGGGTKLTVLGQPKAAPSVTLFP<br>PSSEELQANKATLVCLISDFYPGAVTVAWKADSSPVKAGVETTTPSKQSNNKYAASSYLSLT<br>PEQWKSHRSYSCQVTHEGSTVEKTVAPTECS

Table S2. Equilibrium K_D values from biolayer interferometry studies. Related to Figures 2 and 3.

Treatment	Equilibrium K_D (nM)			Figure
	hIL-2	hIL-2Rα	hIL-2Rβ	
F5111 Ab	2.7	-	-	2A, S1C, S1D
hIL-2	-	16	480	2A, S1C, S1D
Control IC	-	3.4	4.4	2A
hIL-2/F5111 Complex	4.8	18	>2000	2A
F5111 IC LN35	>2000	3.0	>2000	2A
hIL-2	-	25	470	3B, S3B, S5C
Control IC	-	2.5	4.1	3B, S3B
F5111 IC	>2000	3.2	>2000	3B, S3B, S4A
Y33A IC	>2000	2.6	>2000	3B, S3B
Y94A IC	>2000	2.2	>2000	3B, S3B
S96A IC	>2000	2.5	>2000	3B, S3B
Y35A IC	>2000	2.5	>2000	3B, S3B
Y52A IC	>2000	2.8	500	3B, S3B
Y54A IC	>2000	2.8	>2000	3B, S3B
Y60A IC	>2000	2.7	>2000	3B, S3B
V103A IC	>2000	2.4	>2000	3B, S3B
F5111.2 IC	>2000	2.4	>2000	3B, S3B
F5111 IC LN15	>2000	7.5	210	S1C
F5111 IC LN25	>2000	3.4	>2000	S1D
F5111 Ab	2.1	-	-	S1E
hIL-2	-	16	310	S1E
F5111 IC LN25 P1	>2000	11	>700	S1E
F5111 IC LN25 P2	>2000	8.1	>1000	S1E
F5111 IC LN25 P3	>2000	4.8	>2000	S1E
Control IC + N297A	-	3.8	7.7	S4A, S5C
F5111 IC + N297A	>2000	3.6	>2000	S4A, S5C
F5111.2 Ab + N297A	4.9	-	-	S5C
hIL-2/F5111.2 Complex	5.6	68	>2000	S5C

Table S3. EC_{50} and E_{Max} Values from YT-1 cell activation studies. Related to Figure 2.

Treatment	EC_{50} (nM)		Normalized E_{Max}		Figure
	IL-2Rα^{+}	IL-2Rα^{-}	IL-2Rα^{+}	IL-2Rα^{-}	
**hIL-2**	0.13	0.88	82	69	2B, S1F, S1G
**Control IC**	0.031	0.83	87	91	2B
**hIL-2/F5111 Complex**	0.081	1.8	85	62	2B
**F5111 IC LN35**	0.70	>2000	80	21	2B
**hIL-2**	-	0.98	-	94	S1B
**hIL-2:F5111 = 1:1**	-	0.91	-	88	S1B
**hIL-2:F5111 = 1:2**	-	0.89	-	74	S1B
**hIL-2:F5111 = 1:4**	-	0.35	-	64	S1B
**hIL-2:F5111 = 1:7.5**	-	1.7	-	60	S1B
**F5111 IC LN15**	0.10	13	76	65	S1F, S1G
**F5111 IC LN25**	0.58	>2000	84	23	S1F, S1G
**hIL-2**	-	0.57	-	83	S1H (top)
**F5111 IC LN15 P1**	-	8.9	-	75	S1H
**F5111 IC LN15 P2**	-	6.9	-	87	S1H
**hIL-2**	-	0.68	-	81	S1H (bottom)
**F5111 IC LN25 P1**	-	37	-	73	S1H
**F5111 IC LN25 P2**	-	69	-	82	S1H
**F5111 IC LN25 P3**	-	>2000	-	76	S1H

Table S4. EC_{50} and E_{Max} values from human PBMC activation studies. Related to Figures 2, 3, and 5.

Treatment	EC_{50} (pM)			E_{Max} (pSTAT5 MFI)			Figure
	Treg	$CD8^+$ T	T_{Conv}	Treg	$CD8^+$ T	T_{Conv}	
**hIL-2**	4.5	1200	110	5300	3600	2500	2C
**Control IC**	1.3	1600	30	5200	3300	2400	2C
**hIL-2/F5111 Complex**	3.7	240	70	5100	2200	2200	2C
**F5111 IC LN35**	1700	6.1e4	3.7e4	5200	760	1100	2C
**hIL-2**	5.2	1000	110	5300	3500	2400	3C
**Control IC**	1.4	1500	39	5600	3600	2500	3C
**F5111 IC**	2100	5.0e4	3.4e4	5500	740	980	3C
**Y33A IC**	81	2.2e4	1.5e4	5300	1100	1400	3C
**Y94A IC**	6.9	4300	1200	5400	1800	1900	3C
**S96A IC**	910	3.3e4	2.6e4	5100	710	1000	3C
**Y35A IC**	7.7	4100	1400	5700	1800	2100	3C
**Y52A IC**	2.9	3500	360	5500	2300	2200	3C
**Y54A IC**	7.8	3000	1300	5500	1600	1900	3C
**Y60A IC**	15	5300	2900	5400	1400	1800	3C
**V103A IC**	51	1.3e4	7400	5300	1000	1400	3C
**F5111.2 IC**	8600	>2.0e5	>2.0e5	4700	370	460	3C
**hIL-2**	1.8	890	35	3700	2800	2100	5A
**Control IC + N297A**	0.87	2900	19	3600	2700	2000	5A, S4B
**F5111 IC + N297A**	1500	1.8e4	1.8e4	3800	400	770	5A, S4B
**hIL-2/F5111.2 Complex**	8.2	190	140	3700	570	1100	5A
**F5111 IC w/o N297A**	1700	3.2e4	3.4e4	3600	380	700	S4B
**hIL-2**	1.9	660	84	4800	3400	2800	S4C
**Control IC + N297A**	0.48	1200	16	4700	3200	2600	S4C
**Y60A IC + N297A**	17	6300	2900	4900	1100	1500	S4C
**V103A IC + N297A**	42	1.3e4	7200	4800	950	1400	S4C
**Y33A IC + N297A**	47	1.5e4	8000	4900	1000	1500	S4C
**F5111 IC + N297A**	440	1.8e4	7900	4900	790	1200	S4C
**F5111.2 IC + N297A**	1800	1.3e4	1.1e4	4300	310	430	S4C

Table S5. EC_{50} and E_{Max} values from mouse splenocyte activation studies. Related to Figures 3 and 4.

Treatment	EC_{50} (pM)			E_{Max} (pSTAT5 MFI)			Figure
	Treg	$CD8^+$ T	T_{Conv}	Treg	$CD8^+$ T	T_{Conv}	
**hIL-2**	6.8	1.3e5	-	8600	5700	1000	S4D
**Control IC + N297A**	3.2	2.9e5	-	8600	4900	1100	S4D
**Y60A IC + N297A**	93	>2e6	-	8400	280	650	S4D
**V103A IC + N297A**	260	>2e6	-	8000	370	720	S4D
**Y33A IC + N297A**	270	>2e6	-	8100	340	680	S4D
**F5111 IC + N297A**	2100	>2e6	-	7700	320	720	S4D
**F5111.2 IC + N297A**	6300	>2e6	-	5500	220	660	S4D
